# *Caenorhabditis elegans* in nanosafety research: dual effect of *Escherichia coli* ecocorona formation on single and combined toxicity of graphene oxide

**DOI:** 10.1039/d6na00311g

**Published:** 2026-08-06

**Authors:** Francine Côa, Mathias Strauss, Cassio Hamilton Abreu-Junior, Fabrício de Souza Delite, Daiana Silva de Ávila, Beatriz Santana Borges, Rodrigo Villares Portugal, Iseult Lynch, Diego Stéfani Teodoro Martinez

**Affiliations:** a Brazilian Nanotechnology National Laboratory (LNNano), Brazilian Center for Research in Energy and Materials (CNPEM) Campinas São Paulo Brazil francinecoa@gmail.com i.lynch@bham.ac.uk diego.martinez@lnnano.cnpem.br; b Center for Nuclear Energy in Agriculture (CENA), Universidade de São Paulo (USP) São Paulo Brazil; c Grupo de Pesquisa em Bioquímica e Toxicologia em Caenorhabditis elegans (GBToxCe), Universidade Federal do Pampa (UNIPAMPA) Uruguaiana Rio Grande do Sul Brazil; d School of Geography, Earth and Environmental Sciences, University of Birmingham Edgbaston B15 2TT Birmingham UK; e Centre for Environmental Research and Justice, University of Birmingham Edgbaston B15 2TT Birmingham UK

## Abstract

*Caenorhabditis elegans* is a free-living nematode widely applied in nanosafety research. It feeds primarily on organic matter, with bacteria as its primary nutrient source. Under experimental conditions, *Escherichia coli* is routinely provided as food to prevent starvation in *C. elegans*. However, its influence on *C. elegans*' biological responses to nanomaterials remains unexplored. This study investigated the implications of *E. coli* ecocorona formation on the physicochemical properties, colloidal behaviour and toxicity of graphene oxide (GO), both individually and combined with silver ions (Ag^+^), in the *C. elegans* model. Using a multi-technique approach, we observed significant changes in GO's topography, surface roughness, chemical composition, and colloidal stability, indicating that *E. coli* biomolecules coated the GO surface selectively. Notably, the ecocorona mitigated GO toxicity, as it suppressed lethality and neuronal injuries induced by GO while also reducing adverse effects on the germline and hindering the direct contact of GO with the intestinal barrier. In contrast, the combination of ecocorona-coated GO (EC@GO) with Ag^+^ aggravated Ag^+^ lethality by 460%, and was the most damaging treatment to neurons and reproductive organs. Organisms exposed to EC@GO with Ag^+^ accumulated twice as much Ag as those exposed to bare GO with Ag^+^, indicating that the ecocorona acted as a carrier of silver. These outcomes provide the first evidence of the dual effect of the *E. coli* ecocorona in modulating the individual and combined toxicity of GO, underscoring the importance of the *C. elegans* research community implementing routine characterization of the *E. coli* ecocorona as a prerequisite in nanosafety assessments.

## Abstract 2

*Escherichia coli* ecocorona formation drives distinct toxicological responses to graphene oxide alone and in combination with metals in *Caenorhabditis elegans*.

## Graphical abstract

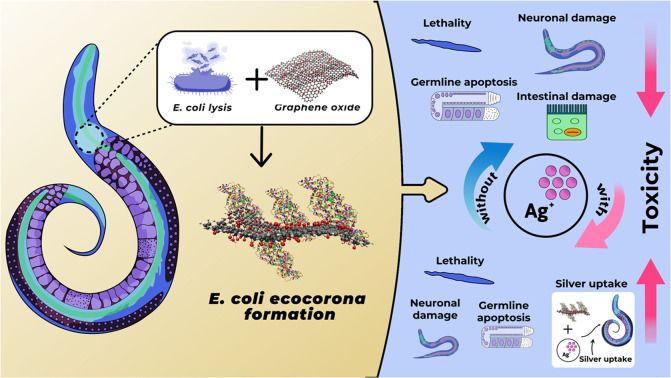

## 1. Introduction

Around 60 years ago, Sydney Brenner proposed the free-living nematode, *C. elegans*, as an ideal system for molecular biology studies due to its notable genomic homology with humans (about 41.7%) and similarities in physiology, anatomy, and metabolism.^1^ Some decades later, *C. elegans* became a pivotal model organism to investigate the uptake, distribution, excretion and adverse effects of nanomaterials (NMs) because of its multicellular complexity, high sensitivity to environmental changes, short life cycle, high reproductive rate, small size, transparency and the broad availability of transgenic strains that allows a detailed investigation of toxicity mechanisms and molecular pathways.^2,3^

Graphene oxide (GO) is a versatile carbon nanomaterial widely applied in electronics, composites, sensors, energy capture and storage, and water remediation.^4^ In the biomedical field, GO has attracted considerable attention for applications such as drug delivery, tissue engineering, biosensing, and bioimaging. However, the increasing development of GO-based technologies has also raised concerns regarding their potential impacts on human and environmental health, driving extensive efforts to understand their safety profile.^5^ Studies with *C. elegans* have provided valuable insights in this direction, indicating that GO can affect the growth, lifespan, reproduction, and fertility of *C. elegans*, besides inducing oxidative stress, germline apoptosis and neurotoxicity.^6–11^ These adverse effects have been associated with GO's physicochemical properties.^6,10,12^ However, a few studies have noticed that exposure conditions may modulate the GO toxicity.^13^

Experiments with *C. elegans* and NMs are typically performed in a range of experimental conditions, including liquid or solid media with varying compositions and ionic strengths, with and without the addition of food.^3,13^ This lack of adoption of standardized methodologies makes data integration, meta-analysis and predictive modelling challenging because the extrinsic properties of NMs, which depend on their surroundings including pH, ionic strength, presence of biomolecules, can cause unexpected experimental artifacts.^14^ A critical aspect is the addition of a food source, usually the bacterium *E. coli*.^15^ Despite preventing nematode starvation, *E. coli* may significantly interact with NMs, influencing their behaviour and toxicity in different ways. Bacteria may either agglomerate with some NMs, modifying their availability and toxicity,^16^ or they may be concomitantly ingested with NMs by nematodes.^17^ Once ingested, *E. coli* cells are ground in the pharynx and the lysed bacterial content is transferred to the *C. elegans* intestine, where the internalized NMs may be retained. The gut of *C. elegans* is exposed to the NMs and the reactive surface of NMs drives them to interact with their surroundings and bind to the newly available biomolecules from lysed bacterial content.^13,18^ This binding of biomolecules leads to the formation of a coating on the NMs surface, referred to in this study as the *E. coli* ecocorona (EC).

It is well established that NMs adsorb various biomolecules when exposed to biological fluids or environmental matrices.^19–21^ For GO, previous studies have shown that the adsorption of proteins onto its surface can alter its colloidal stability, cellular interactions, and toxicity, often reducing direct interactions with biological membranes and modifying cellular uptake.^22–24^ These findings highlight that GO toxicity is not solely determined by its intrinsic physicochemical properties, but also by the identity of the biomolecules associated with its surface. Although substantial efforts have been directed to understand ecocorona formation in other contexts, this phenomenon remains largely unexamined in *C. elegans* nanosafety research, limiting our understanding of nano-eco interactions, particularly how nanomaterials interact with environmentally derived biomolecules and how these interactions influence biological responses and toxicological outcomes in exposed organisms. Beyond ecocorona formation, NMs can interact with other substances or copollutants present in the environment, such as heavy metals or organic pollutants.^25^ Wang *et al.* (2018), for example, reported that TiO_2_ nanoparticles interacted with arsenic, cadmium, and nickel, increasing their bioaccumulation and reproductive toxicity toward *C. elegans*.^26^

Although previous studies have evaluated GO toxicity in the presence of bacterial food sources, the specific contribution of an *E. coli*-derived ecocorona to GO toxicity has not been investigated furthermore, it remains unclear if this coating affects the combined toxicity of GO with other co-pollutants. In our study, we hypothesized that *E. coli* ecocorona formation could influence these aspects. Nematodes were exposed to bare and ecocorona-coated GO, with and without silver ions (Ag^+^) and biological endpoints, including survival, germline apoptosis, and neuronal damages, were evaluated. To gain further insights into the NM uptake, accumulation, and resulting intestinal damage, we employed confocal Raman spectroscopy and transmission electron microscopy, while ICP-MS was used to quantify Ag internalization. Our findings provide the first evidence that the *E. coli* ecocorona plays a crucial role in determining GO toxicity, modulating its individual and combined toxic effects in *C. elegans*.

## 2. Experimental section

### 2.1. Materials

Graphene oxide was prepared according to a modified Hummers' method, as described by Côa *et al.* (2022).^6^ Briefly, GO was obtained by the oxidative exfoliation of graphite flakes with sulfuric acid and potassium permanganate. After synthesis, GO was freeze-dried for storage. Before the experiments, a GO stock-dispersion (0.5 mg mL^−1^) was prepared following the OECD Test Guideline No. 318 (OECD, 2017) and the protocol established by Côa *et al.* (2022).^6^ In brief, 10 mg of GO was pre-wetted with 200 µL of ultrapure water, followed by the addition of 19.8 mL ultrapure water after 24 h. The dispersion was then subjected to bath sonication (Cole-Parmer, model 08895-43, USA) for 80 min at a controlled temperature (15–20 °C). Ultrapure water from a Milli-Q Millipore system (Millipore, USA) was used to prepare all solutions.

Silver nitrate (AgNO_3_, Lot 46624, 99.93% purity, Neon Company, Brazil) was used to prepare a stock solution of silver ions (200 mg L^−1^) in ultrapure water, which was stored at 4 °C in the dark until use.

As recommended by the EU NanoReg project,^27^ moderately hard reconstituted water, referred to as EPA medium, was used in our biological assays as it is a low ionic strength medium. The EPA medium is composed of 60.0 mg L^−1^ CaSO_4_·2H_2_O, 60.0 mg L^−1^ MgSO_4_, 96.0 mg L^−1^ NaHCO_3_ and 4.0 mg L^−1^ KCl (all reagents ≥99.5% purity, analytical grade, Sigma Aldrich, USA)

### 2.2. *E. coli* lysis and ecocorona formation

*E. coli* OP50 strain was obtained from the *Caenorhabditis Genetics* Center (CGC) and maintained on Luria–Bertani (LB) agar plates at 10 °C. Prior to the lysis procedure, OP50 cells were inoculated into LB broth and cultured at 37 °C for 14 h. Bacterial lysis was performed following the procedure summarized below and described in detail in the SI. Briefly, the bacterial suspension was centrifuged, the supernatant discarded, and the resulting pellet rinsed with phosphate-buffered saline (PBS). The suspension was then subjected to sonication (40 W, 10 min) using an ultrasonic tip processor (Model Gex 400, Acil Weber) under temperature control (10 °C), followed by centrifugation. The supernatant, containing soluble biomolecules, was filtered through a 0.22 µm membrane (Millipore, USA) and collected. Protein concentration was determined by the Bradford assay kit (Sigma-Aldrich) (Fig. S1) and adjusted to 1 mg mL^−1^ for further use. The extracted biomolecules were maintained frozen (−80 °C).

To promote the formation of an *E. coli* ecocorona on GO, GO dispersion (200 µL, 0.5 mg mL^−1^) was incubated in suspension with 500 µL of the *E. coli* extracted soluble proteins (1 mg mL^−1^) and 300 µL PBS in 1.5 mL Eppendorf tubes at 37 °C for 60 min using a thermoblock (Thermomixer C, Eppendorf, Germany). Centrifugation and washing steps were performed to remove weakly adsorbed biomolecules. Tubes were centrifuged at 10 000 rpm, 4 °C for 60 min. Supernatants were discarded, and pellets were resuspended in 10× diluted PBS. This washing procedure was repeated three times to obtain the GO-protein complex (*i.e.*, pellet form), hereafter referred to as ecocorona-coated GO (EC@GO). Samples were stored at 10 °C and used within two days. The ecocorona-coated GO was always freshly prepared before each experiment. EC@GO was subsequently used for physicochemical and proteomic characterization, as well as toxicity experiments.

Since the washing and centrifugation steps could reduce the GO concentration in the EC@GO complex, the GO concentration in the pellets was routinely verified before preparing sub-stock solutions for experiments. This step ensured accurate determination of GO content for subsequent use in the experiments. A calibration curve, prepared as described in our previous article^6^ following the protocol of Cerrillo *et al.* (2015),^28^ was used to assess the GO concentration in the pellets by UV-vis spectroscopy at 400 nm.

### 2.3. Physicochemical characterization of bare and ecocorona-coated GO

A multi-analytical approach was applied to study the physicochemical properties of GO with and without the ecocorona. Detailed experimental procedures are provided in the supplementary information.

Atomic force microscopy (AFM) was performed to evaluate the surface morphology, roughness and thickness of GO and EC@GO. GO and EC@GO were deposited (5 µL) onto freshly cleaved mica substrates (2 × 2 cm) and dried in an acrylic chamber under nitrogen flow prior to analysis. AFM images were acquired using a silicon tip probe in tapping mode using a MultiMode VIII microscope equipped with a NanoScope V controller (Bruker, USA). AFM images were processed using Gwyddion software (version 2.5). Plane leveling and background correction were applied prior to quantitative analysis. Height profiles were obtained from cross-sectional analysis of individual flakes (*n* = 20 per sample), and surface roughness was calculated as root-mean-square (RMS) roughness over equivalent flake areas (12 808 nm^2^). Detailed acquisition parameters are provided in the SI.

The surface charge of GO and EC@GO was assessed by electrophoretic light scattering (ELS) at a concentration of 10 mg L^−1^ in EPA medium and NaCl solution (0.1 mM), using the Zetasizer Ultra (Malvern Panalytical, UK). Measurements were performed in triplicate, and results are reported as mean ± standard deviation.

In addition, the surface elemental composition of GO and EC@GO was analysed by X-ray photoelectron spectroscopy (XPS), using a K-Alpha System (Thermofischer Scientific, USA) operated with Al Kα X-rays and charge compensation. Survey spectra were collected with 400 µm spatial resolution at five different areas per sample using a pass energy of 50 eV. Data processing was performed using Thermo Avantage software (version 5.957). Functional groups present on GO and EC@GO surfaces were characterized by attenuated total reflectance Fourier-transform infrared spectroscopy (ATR-FTIR) using an IRSpirit-L (Shimadzu, Japan). Spectra were collected from 500 to 4000 cm^−1^ with a resolution of 2 cm^−1^ and 150 scans per sample.

To better understand ecocorona formation, SDS-PAGE and LC-MS/MS were applied as complementary biochemical techniques to investigate the profile and identity of *E. coli* proteins adsorbed by GO. First, the protein profile was evaluated by SDS-PAGE (4% stacking gel and 15% resolving gel), which separates proteins based on molecular weights. Then, the different proteins that compose the *E. coli* ecocorona were identified by LC-MS/MS. Protein bands were removed from the acrylamide gel and submitted to protein digestion and peptide extraction as described previously.^29,30^ The resulting peptides were purified using the StageTips C18 (prepared in-house) method and analyzed by LC-MS/MS using an LTQ Orbitrap Velos mass spectrometer (ThermoFisher Scientific, Waltham, USA). The entire procedure is described in the SI.

Proteomic data obtained from the GO-associated fraction were compared with the total *E. coli* lysate to identify proteins selectively associated with the ecocorona. The complete proteomic datasets and comparative protein lists are provided in the SI (Tables S2–S5).

### 2.4. Dispersion characterization and colloidal stability studies

For the colloidal stability studies, the amount of GO and EC@GO in suspension, with and without silver ions, was measured by ultraviolet-visible (UV-vis) spectrophotometry.

Dispersions of 10 mg L^−1^ GO and EC@GO were prepared in ultrapure water (UW) and EPA medium to assess the effect of ionic strength on colloidal stability, and aliquoted on a 24-well plate to replicate the conditions of biological assays, as suggested in OECD Test Guidance No. 318 that specifies a procedure to gain information on dispersion stability of NMs in simulated environmental media.^31^ Aliquots of 100 µL were taken from the top of each well at 1, 3, 6, and 24 h, and absorbance at 400 nm was measured in a microplate reader (Multiskan TM GO, Thermo Scientific, USA). Data were expressed as *A*/*A*_o_, where *A* is the value obtained at each time point, and *A*_o_ is the initial absorbance (0 h). Digital images were also captured to assess the material stability visually.

In addition, dynamic light scattering (DLS) measurements were performed to evaluate the hydrodynamic size of GO and EC@GO dispersions in both UW and EPA medium. Analyses were conducted using a Zetasizer Ultra (Malvern Panalytical, UK). For each sample, three independent cuvettes were prepared, and four consecutive measurements were performed per cuvette. Results were expressed as mean values of all measurements. Quality criteria, including correlogram quality, count rate, and measurement repeatability, were considered to ensure data reability.

Moreover, silver speciation under exposure conditions was modelled using chemical equilibrium diagrams generated by the Spana® software.

### 2.5. Silver adsorption by materials

Silver adsorption onto GO and EC@GO was investigated using a fixed concentration of the materials (10 mg L^−1^) and two Ag^+^ concentrations (5 and 15 mg L^−1^). Samples were incubated in EPA medium at 20 °C for 24 h on a rotatory shaker (Luferco Phoenix, model AP-22, Brazil). Then, samples were centrifuged at 14 000 rpm for 1 h at 20 °C. Supernatants were collected and acidified in 5% HNO_3_ (ultrapure grade, Sigma-Aldrich, USA) for further analysis. Silver concentrations in the samples were quantified using inductively coupled plasma mass spectrometry (ICP-MS, Agilent Technologies 7500ce, USA). The adsorption capacity of GO and EC@GO for silver removal was calculated by determining the difference between the initial and final silver concentrations in solution.

### 2.6. Biological experiments

#### 2.6.1. *Caenorhabditis elegans* culturing and synchronization

Wild-type (N2 Bristol) and transgenic strains (MD701 –[*lin-7p::ced-1::GFP + lin-15(+)*] and AML175 –[*wtfIs3 [rab-3p::NLS::GFP + rab-3p::NLS::tagRFP*]]) worms were acquired from the *Caenorhabditis Genetics* Center (CGC), as were the bacteria strains (OP50 and NA22). Worms were maintained at 20 °C on nematode growth medium (NGM) plates seeded with *E. coli* OP50, following the methodology described by Stiernagle (2006).^32^ For synchronization, nematodes were cultured on 8P-agar medium with a lawn of *E. coli* NA22, which was used because it supports higher worm densities and facilitates the production of large populations of age-synchronized nematodes. Age-synchronized worms were isolated from gravid adults using a bleaching solution (1 mL NaOH 10 M, 4 mL NaClO 3%, and 5 mL ultrapure water), followed by rinsing in EPA medium, as recommended by Porta-de-la-Riva *et al.* (2012).^33^ The eggs were then filtered through a 40 µm cell strainer (Corning, USA) to remove worm debris and transferred to NGM plates seeded with *E. coli* OP50 and incubated at 20 °C for 48 h to obtain young nematodes at L3–L4 stage. Prior to all biological assays, age-synchronized worms were transferred from NGM plates to EPA medium and washed three times to remove residual bacteria and bacterial debris originating from the culture plates. After washing, worms were immediately used in the exposure experiments. All biological assays were performed in liquid EPA medium without the addition of food or live bacteria.

#### 2.6.2. Preparation of sub-stocks for toxicity assays

Toxicity assays were conducted with young nematodes exposed in liquid medium (EPA) using 24-well plates. All biological experiments were carried out under static conditions at 20 °C in the dark using a BOD incubator (TE-371, TECNAL, Brazil).

Prior to single exposures, sub-stocks of GO, EC@GO or AgNO_3_ were prepared by diluting the stock dispersions in UW and freshly applied in the toxicity tests. For EC@GO, sub-stocks were prepared based on the GO concentration measured in the pellets after the washing procedure, as described above. The exposure concentrations were defined according to the GO content in both GO and EC@GO treatments, and the mass of adsorbed proteins was not included in the dose calculation, ensuring a consistent and comparable exposure metric between conditions. For co-exposure assays, a fixed and nontoxic concentration of GO or EC@GO (0.1 µg L^−1^) was incubated with increasing concentrations of Ag^+^ in EPA medium. Incubation was carried out for 30 minutes in a rotatory shaker to allow silver binding and speciation changes. Immediately after incubation, the mixtures were applied to the assays.

#### 2.6.3. Lethality assays

Lethality assays were carried out according to the protocol of Maurer *et al.* (2015).^34^ Young adult worms (L3–L4 stage) were exposed to Ag^+^ and materials in 24-well plates for 24 h. For single exposure assays, 15–20 worms (15 µL) were added per well with 100 µL test-solution and 885 µL EPA medium. In co-exposure tests, 200 µL test-solution with 15-20 worms (15 µL) and 785 µL EPA medium were added per well.

Nematodes were exposed to Ag^+^ concentrations ranging from 1.8 to 35.8 µg L^−1^ and to GO and EC@GO at concentrations ranging from 0.0001 to 10 mg L^−1^. In co-exposure tests, a nontoxic concentration of GO or EC@GO (*i.e.*, 0.1 µg L^−1^) was combined with Ag^+^ concentrations ranging from 1.8 to 13 µg L^−1^.

Mortality was measured after 24 h by counting the living worms under a stereomicroscope (Stemi 508, Zeiss, Germany). Worms were considered dead if they did not respond to a stimulus with a metal wire. Survival rates were calculated to generate dose–response curves. Each experiment was conducted at least three times. The concentrations that kill 50% of the organisms (LC_50_) after 24 h of exposure were calculated with 95% confidence intervals (CI) using GraphPad Prism. Dose–response curves were generated using a sigmoidal fitting in Origin Pro 2022b software (Origin Lab, USA).

#### 2.6.4. Biodistribution, internalization and effects on *C. elegans* intestinal barrier

Internalization and biodistribution of GO, EC@GO, GO + Ag^+^, and EC@GO + Ag^+^ in nematodes were investigated using confocal Raman spectroscopy. 60 L4-stage *C. elegans* were exposed to 10 mg L^−1^ GO or EC@GO, and to combinations of these materials at 10 mg L^−1^ with the LC_50_ values obtained for GO + Ag^+^ and EC@GO + Ag^+^. After 48 h of exposure, nematodes were washed twice with EPA medium by centrifugation at 2100 rpm, 20 °C for 3 min, and preserved in 2% paraformaldehyde (Lot #SLBF2268V, analytical grade, Sigma-Aldrich). After fixation, nematodes were deposited onto a standard glass microscope slide in a minimal volume of liquid and analysed without a coverslip. The experiment was replicated twice. At least five nematodes per treatment were randomly collected for analysis in a confocal Raman spectrometer (XploRA PLUS, Horiba, Japan) equipped with an optical confocal microscope (50× objective). Raman spectra were acquired over *z*-depth from −30 to 120 µm (with the upper cuticle as the 0 µm reference) using 5 µm as acquisition steps. Each spectrum was recorded from 800 to 2000 cm^−1^, with 6 accumulations of 3 s each, using a 532 nm excitation wavelength and a 1.2 mW laser power.

Due to the physicochemical properties of GO, including its tendency to aggregate and interact with biological surfaces, a fraction of the material is expected to remain adhered to the nematode cuticle or present in the surrounding medium even after washing procedures. Therefore, the results were analysed considering this aspect, and the interpretation of the data was discussed in detail in the Results section.

The impact of material exposure on *C. elegans* microvilli was studied by applying transmission electron microscopy. Nematodes were fixed in Karnovisky's solution (2.5% glutaraldehyde; 4% paraformaldehyde in 0.1 M CaCO_3_ buffer) for 1 h after exposure to GO and EC@GO at 10 mg L^−1^, and to the LC_50_ values of GO + Ag^+^, and EC@GO + Ag^+^. Samples were post-fixed for 1 h in 1% osmium tetroxide, followed by dehydration steps through a graded acetone series (30%, 50%, 70%, 90%, 100% twice) for 10 min each step. Dehydrated samples were then embedded in Embed 812 resin (Electron Microscopy Sciences, EMS®, USA). An RMC Boeckeler ultramicrotome was applied to cut the samples into sections of 70 nm, which were contrasted with 2% uranyl acetate and 1% lead citrate. Imaging was performed on a 200 kV Talos Arctica Transmission Electron Microscope (TEM, Thermo®).

#### 2.6.5. Silver internalization by nematodes

The total silver concentration in *C. elegans* was evaluated by ICP-MS. To avoid undesired silver contaminants, all borosilicate glassware was acid-cleaned by soaking in aqua regia (a mixture of three parts hydrochloric acid and one-part nitric acid), followed by two rinses with tap water and five rinses with ultrapure water. Metal-free plastics were sourced from SSIbio and BioClean.

2700 L4-stage worms (equivalent to 1 mL of worms suspension) were transferred into 50 mL centrifuge tubes containing 5 mL of the test solution and 44 mL of EPA medium. Worms were exposed to the LC_50_ concentrations for each treatment: 12.4 µg L^−1^ Ag^+^, 0.1 µg L^−1^ GO + 5.7 µg L^−1^ Ag^+^ and 0.1 µg L^−1^ EC@GO + 2.7 µg L^−1^ Ag^+^. Six independent replicates were prepared for each treatment group. To ensure accuracy, ten procedural blanks (with and without nematodes – only reagents) were included during sample preparation.

After 24 h of exposure, worms were immobilized by cooling to 5 °C. Then they were centrifuged and pelleted at 5000 rpm, 4 °C for 5 min. Supernatants were discarded to remove residual exposure media, and the resulting pellets (1 mL) were transferred to 1.5 mL Eppendorf tubes. To remove residual Ag^+^, worms underwent three cycles of centrifugation and washing with ultrapure water. Pelleted worms (200 µL) were transferred to 15 mL centrifuge tubes, and completely digested with 50% HNO_3_ (Lot 385212, Sigma Aldrich, purified in a SubClean sub-boiling distillation system from Milestone, Italy). This was achieved by adding 800 µL of 62.5% HNO_3_ to the samples. Samples were digested overnight at room temperature (20 °C), then diluted to a final volume of 10 mL with ultrapure water to achieve a 5 vol% nitric acid concentration.

ICP-MS measurements were carried out on an Agilent Technologies 7500ce ICP-MS. A calibration curve was prepared using standards at concentrations of 0.1; 1, 10; 100, and 200 µg L^−1^ Ag^+^, derived from an environmental calibration standard (Lot 13-166JB, 5% HNO_3_, Agilent Technologies) composed of Ag, Al, As, Ba, Be, Co, Cr, Mn, Nb, and Ni. The calibration curve exhibited a suitable coefficient for analyses (*r*^2^ = 0.9999). The silver content internalized by the nematodes was calculated by dividing the total mass of silver detected by the number of digested worms, which was counted following a standardized protocol described by Scanlan *et al.* (2018).^35^ The percentage of internalized silver was calculated in relation to the initial total silver concentration to which the nematode was exposed (*i.e.*, LC_50_ values). Each experiment was performed in duplicate.

#### 2.6.6. Neuronal effects assessment

L4-stage worms of the AML175 strain (expressing the calcium insensitive fluorescent proteins GFP and tagRFP in the nuclei of all neurons in a lite-1(ce314) background) were exposed to single and co-exposure conditions, similar to the lethality experiments. After 24 h, worms were transferred to 1.5 mL Eppendorf tubes and centrifuged at 2100 rpm, 20 °C for 3 min. Then, they were mounted on microscope slides, anaesthetized with 6 mM levamisole (Lot 05-096/03, Noxon) and covered with coverslips. Fluorescence imaging was performed in an inverted stereomicroscope (Axio Vert.A1, Zeiss, Germany) equipped with an Axion 203 camera, fluorescence module, and an FITC filter set (excitation/emission: 495 nm/519 nm). Image acquisition parameters such as exposure time, gain, saturation, and colour gain were standardized using the Zeiss software to ensure consistency across the samples. Experiments were repeated twice. At least 60 random worms per treatment were analysed. Relative fluorescence intensity of the worm body was quantified using ImageJ software (version 1.52a). Fluorescence images were converted to 8 bit grayscale, and a region of interest encompassing the entire worm body was manually defined. Relative fluorescence intensity was calculated as the mean fluorescence intensity within the ROI for each worm. Results were expressed as a percentage relative to the control group (non-exposed).

#### 2.6.7. Germline cell apoptosis evaluation

Apoptotic germline cells were measured using MD701 transgenic worms, which express CED-1::GFP in the nuclei of gonadal sheath cells (Lant and Derry, 2013),^36^ enabling the visualization of apoptotic germ cells. In this assay, L4-stage worms were exposed for 24 h to the materials, with and without silver ions, in 24-well plates. In the single exposure experiments, worms were exposed to GO and EC@GO at concentrations of 0.0001; 0.01; 1 and 10 mg L^−1^. In co-exposure assays, worms were treated with Ag^+^ at concentrations of 2.7, 5.7, and 12.4 µg L^−1^, either alone or in combination with 0.1 µg L^−1^ of GO or EC@GO. After exposure, worms were transferred to 1.5 mL Eppendorf tubes, rinsed with EPA medium, and placed onto microscope slides with 10 µL of 2 mM levamisole to immobilize them. Slides were sealed with cover glass for imaging. Apoptotic cells in the gonad loop region were analysed using an Olympus microscope (BX-53, Japan) equipped with a 60× objective, a camera, fluorescence module, and an FITC filter set. At least 10 worms per treatment group were evaluated. Each treatment was independently replicated four times, and all experiments were performed in duplicate to ensure reliability.

#### 2.6.8. Data analysis and statistics

Data are expressed as means with standard error of the mean (SEM). Prior to statistical analysis, data normality and homogeneity of variances were assessed using the Shapiro–Wilk and Bartlett's tests, respectively. Statistical comparisons were then performed using one- or two-way analysis of variance (ANOVA) followed by Tukey's post hoc test for multiple comparisons. Analyses were conducted in GraphPad Prism (version 5.0), and results with *p*-values <0.05 were considered statistically significant.

## 3. Results and discussion

### 3.1. Characterization of *Escherichia coli* ecocorona formation

**Fig. 1:**
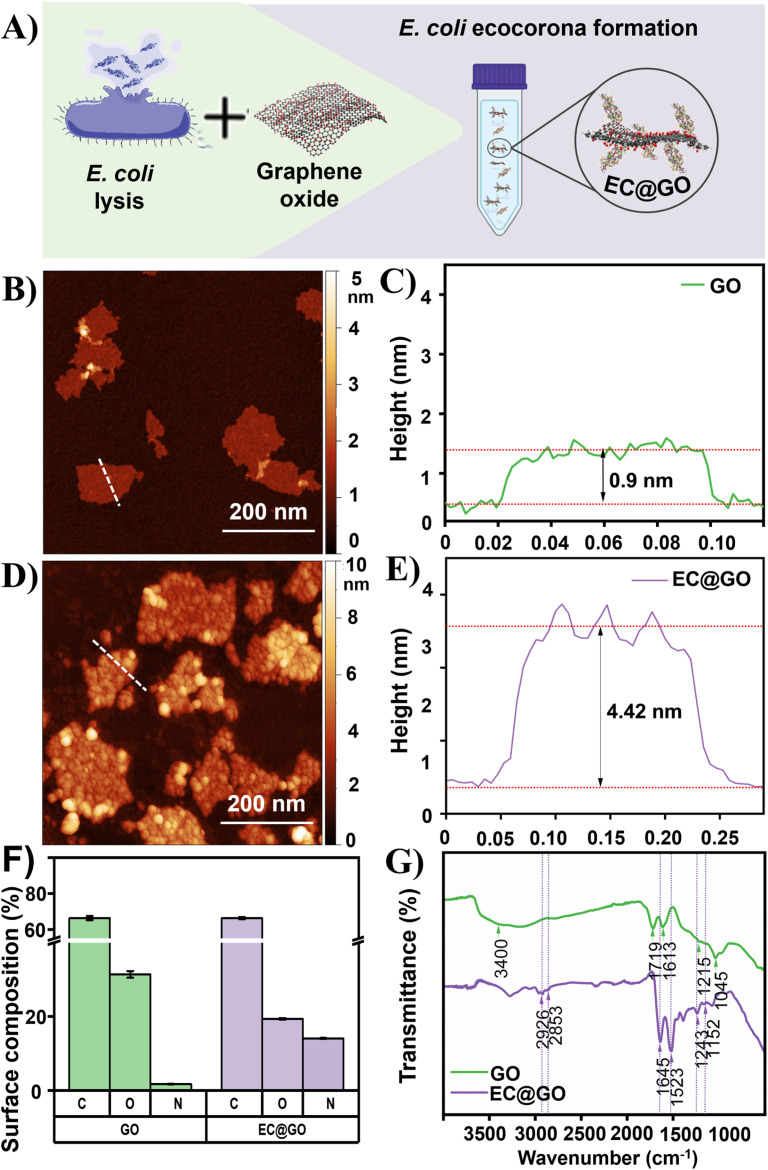
Graphene oxide characterization before and after its interaction with *E. coli* biomolecules. (A) Schematic representation of the *E. coli* ecocorona formation on GO. (B) AFM image of GO and (D) EC@GO and their corresponding height profiles (C and E, respectively). (F) Surface elemental composition of GO and EC@GO determined by XPS analysis. (G) Attenuated total reflectance-Fourier transform infrared spectroscopy (ATR-FTIR) data. Purple dotted vertical lines indicate protein-associated bands that emerged after ecocorona formation.

The formation of biomolecular coatings on the surface of nanomaterials affects their physicochemical properties, colloidal stability, and toxicological profile.^37^ Therefore, applying a multi-technique approach is crucial to understand the changes induced by biomolecule adsorption. Our study adopted methods aligned with current best practices for characterizing ecocoronas.^38^Fig. 1 shows the properties of GO before and after ecocorona formation. The complex GO-ecocorona was named ecocorona-coated GO, or EC@GO.

AFM is a powerful technique to study nano-biomolecule interfaces because it allows direct measurement of topographic changes at the nanoscale.^39^ In our investigation, AFM images reveal globular-like structures distributed across the GO surface, evidencing that its morphology was modified by the adsorption of *E. coli* biomolecules, resulting in a biomolecular coating on the GO flakes (Fig. 1B and D). This finding is further supported by an increase in surface roughness, as the surface of EC@GO became rougher (1.02 ± 0.4 nm) than that of bare GO (0.25 ± 0.3 nm).

Height profiles extracted from AFM images (Fig. 1C and E) also demonstrate an increase in flake thickness after biomolecule interaction. Pristine GO exhibited thickness values of 1.02 ± 0.45 nm, consistent with few-layer graphene oxide, whereas EC@GO exhibited higher thickness values (4.22 ± 1.5 nm). Quantitative analysis of flake thickness (*n* = 20) further confirms the increase in EC@GO compared with bare GO and reveals a broader distribution of thickness values for EC@GO, as evidenced in a boxplot (Fig. S2). These outcomes suggest that biomolecules have effectively coated the GO surface, forming a distinct ecocorona layer on the flakes, characterized by heterogeneous thickness and localized clustering.

In addition to AFM, X-ray photoelectron spectroscopy (XPS) provides valuable insights into ecocorona formation since it is highly sensitive in analyzing the NM surface elemental composition.^40,41^ Our XPS survey data demonstrate that bare GO is composed of 66.4 ± 1.2% carbon, 31.4 ± 0.9% oxygen and 1.7 ± 0.1% nitrogen atoms, whereas EC@GO presents 66.3 ± 0.6% carbon, 19.4 ± 0.2% oxygen and 14.1 ± 0.1% nitrogen (Fig. 1F). This increase in nitrogen abundance observed for EC@GO can be ascribed to the protein adsorption on the GO surface, as proteins are nitrogen-rich compounds.^42^

Previous studies have demonstrated that protein attachment on NMs can be identified by attenuated total reflectance-Fourier transform infrared spectroscopy (ATR-FTIR).^43,44^ ATR-FTIR is useful because it enables the identification and characterization of the diverse functional groups present on NM surfaces, thereby enhancing our understanding of protein-NM interactions. Comparing the FTIR spectra of GO and EC@GO (Fig. 1G), an increased diversity of functional groups on the GO surface after biomolecules interaction is apparent, consistent with findings from other studies of ecocorona formation.^44,45^ In the GO spectrum, peaks at 1045, 1215, 1613, 1719, and a broad band at 3400 cm^−1^ correspond to C–O (primary alcohol) stretching vibration, C–O (epoxy) and C–OH (phenol), C=C (aromatic carbon bonds), C=O (carboxylic acid and carbonyl moieties), and O–H stretch vibration, respectively.^46,47^ By contrast, the FTIR spectrum of EC@GO displays a decrease in intensities of oxygen-related peaks (at 1718 and 1045 cm^−1^) and the emergence of nitrogen-group containing peaks; two strong signals, at 1645 and 1523 cm^−1^, associated with amide groups (N–C=O), indicate amide linkage formation, suggesting that carboxylic groups on GO have reacted with NH_2_ groups from *E. coli* proteins.^48,49^ Moreover, a peak corresponding to aromatic amide groups (C–N) appears at 1243 cm^−1^,^40,41^ along with –CH_3_ and –CH_2_ stretching vibrations at 2853 and 2926 cm^−1^, which are related to the amino acids that compose proteins.^50^ The presence of phosphate groups at 1152 cm^−1^ further supports the binding of proteins to GO.^51^ Together, the FTIR and XPS data suggest the formation of a GO-protein complex since they proved the attachment of nitrogen-related groups on the GO surface. It is worth mentioning that these observed functional groups (*i.e.*, oxygen and nitrogen-related, mainly carboxylates, amines and amides) will be mainly responsible for facilitating interactions with co-exposed pollutants because they work as active sites to adsorb, for example, heavy metal ions.^52,53^

Along with these surface modifications, zeta potential measurements provide additional evidence of ecocorona formation. Pristine GO exhibited a surface charge −48.7 ± 1.2 mV, which became less negative (−31.5 ± 0.8 mV) upon interaction with biomolecules. This variation reflects modifications in the surface electrostatic environment associated with the presence of the biomolecular layer, which may influence interparticle interactions and colloidal stability. Zeta potential values are summarized in Table S1.

**Fig. 2:**
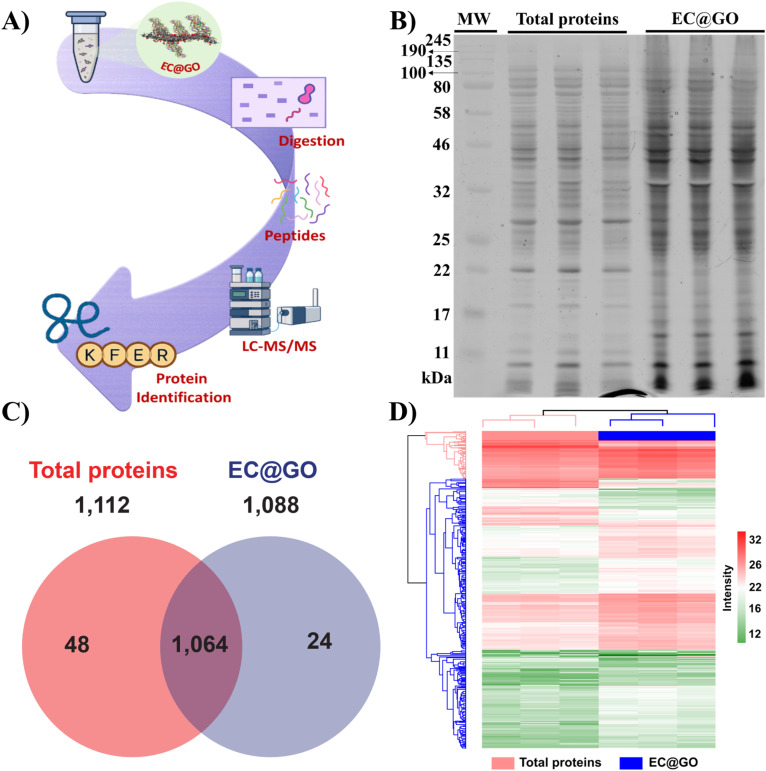
Biochemical characterization of the *E. coli* ecocorona associated with the GO surface. (A) Schematic representation illustrating the formation of the *E. coli* ecocorona on GO and the subsequent SDS-PAGE and LC-MS/MS analyses. (B) SDS-PAGE distribution pattern of total proteins from the *E. coli* lysate (control) and those that were adsorbed onto GO (EC@GO). (C) Venn diagram displaying the number of identified proteins on the *E. coli* lysate and GO surface from LC-MS/MS analysis. (D) Heat map of the top 567 most abundant proteins, clustered in two groups: *E. coli* lysate (pink) and EC@GO (blue). The heat map shows three replicates for each sample (with excellent reproducibility), with each row representing a single identified protein.

The binding affinity between GO and biomolecules involves different mechanisms, including the types of functional groups present on the GO surface, van der Waals interactions, covalent bond formation, and π–π stacking. These mechanisms may co-exist during the protein adsorption, but one may prevail over others, resulting in preferential adsorption of specific proteins.^54^ Sodium dodecyl sulfate-polyacrylamide gel electrophoresis (SDS-PAGE) is a valuable technique for qualitatively identifying proteins adsorbed onto NMs based on their molecular weight.^55^ Using this method, we analysed the profile of total proteins that compose the *E. coli* lysate and compared it with the pattern of proteins adsorbed by GO (Fig. 2A).

The PAGE gel (Fig. 2B) reveals that GO adsorbed a substantial portion of the *E. coli* proteins. However, this adsorption was selective, as the intensity of proteins recovered from GO partially differs from that of the total proteins. In addition, some non-abundant proteins found in the *E. coli* lysate display a high affinity for the GO surface and become greatly enriched on the GO surface, particularly those with molecular weights between 11 and 25 kDa.

In addition to SDS-PAGE analysis, liquid chromatography-tandem mass spectrometry (LC-MS/MS) offers a robust approach for comprehending the characteristics of the ecocorona formed on NMs surfaces. This technique enables precise identification and quantification of proteins through their molecular weight and characteristic peptide mass fingerprints.^56^ The proteins eluted from the GO ecocorona were digested, and the resulting peptides were analysed *via* LC-MS/MS. The complete list of proteins identified in the *E. coli* lysate (1112) and in the *E. coli* ecocorona (1088) is provided in the supplementary material (Table S2). The reliability of the datasets was supported by high Pearson correlations values (>0.8).

As depicted in the Venn diagram (Fig. 2C), 1064 proteins identified in the *E. coli* lysate were also detected in the EC@GO sample, indicating that a large fraction of proteins interacted with the GO surface. However, 48 proteins detected in the lysate dataset were not detected in the EC@GO proteomic dataset. These proteins, are listed in Table S3. Conversely, 24 proteins were detected exclusively in the EC@GO proteomic dataset (Table S4). This observation likely reflects enrichment of specific low-abundance proteins of the lysate during ecocorona formation on the GO surface, which enabled their detection in the recovered EC@GO fraction. Similar enrichment effects have been widely reported in protein corona studies, where adsorption onto nanomaterial surfaces concentrates specific proteins and alters their apparent abundance in proteomic analyses.^30^

To further investigate how protein adsorption onto GO affects the composition of the ecocorona, the relative abundance profiles of the identified proteins were compared. The 567 most abundant proteins detected across the datasets are listed in Table S5 and arranged in a heat map to visualize variations in protein abundance. These proteins were grouped into two clusters (total proteins and EC@GO), which are composed of 84 abundant proteins from the *E. coli* lysate and 482 from the ecocorona. A high-resolution version of the heat map was provided in the SI (Fig. S3).

As shown in Fig. 2D, the abundance profile of proteins present in the GO surface differs significantly from those in the lysate, with a greater abundance of proteins in the ecocorona. The dendrogram further reveals a highly complex relationship between the proteins (*i.e.*, > 10 levels of clustering) that compose the *E. coli* ecocorona, indicating that protein adsorption is not random but governed by selective affinities between proteins and the nanomaterial surface. Such selective adsorption is consistent with previous studies that demonstrated how the surface chemistry of GO strongly influences protein binding.^57,58^ For example, Wei *et al.* (2015)^57^ showed that the degree of reduction modulates the specificity of serum protein interactions, demonstrating that surface chemistry may govern selective adsorption. In agreement with this, Tan *et al.* (2013)^58^ reported that chemical functionalization of GO creates unique interfaces that promote selective adsorption and clustering of protein classes. Together, these findings highlight that the ecocorona composition is governed by both the intrinsic properties of the proteins and the structural/chemical characteristics of the nanomaterial, which may have important implications for how GO is biologically recognized.

The comprehensive morphological, chemical, and biochemical analyses conducted in our study provide strong evidence for the formation of a biomolecular coating on the GO surface, driven by its interaction with *E. coli* lysate. This coating not only increased the roughness and thickness of GO but also introduced nitrogen-containing functional groups on its surface. Proteomic analysis revealed selective protein binding on GO, with an enrichment of low molecular weight proteins (<40 kDa) on its surface. These findings underscore that ecocorona formation substantially modifies the physicochemical identity of GO. Such changes may influence how the nanomaterial interacts with biological systems and therefore may play an important role in determining its toxicological behavior. To further explore this aspect, biological assays were performed to assess whether the *E. coli* ecocorona exerts any effect on the GO toxicity, either individually or in combination with Ag^+^.

### 3.1. The determining role of the *E. coli* ecocorona in mitigating GO toxicity

**Fig. 3:**
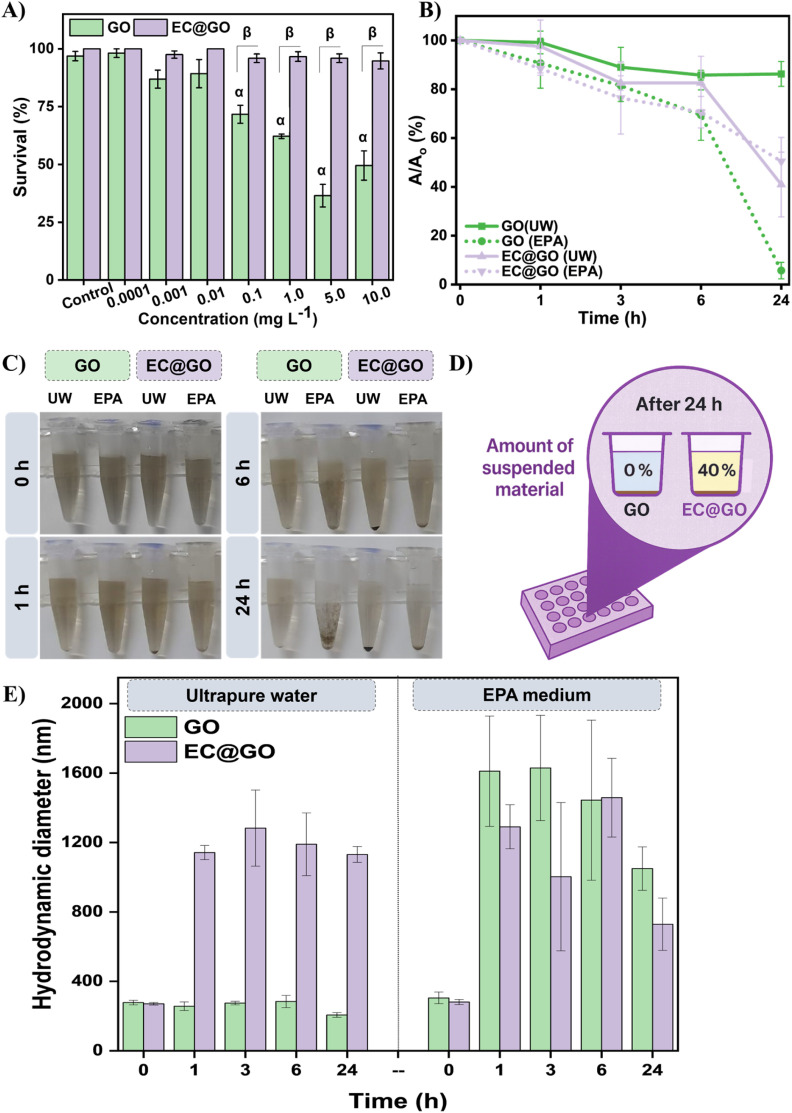
*E. coli* ecocorona effect on GO toxicity and its implication for colloidal stability. (A) Effects of GO and EC@GO (from 0.0001 to 10 mg L^−1^) on nematode survival. Bars indicate the mean with the standard errors. Significant differences between the control group and different treatments are indicated by α, while statistically significant differences among the treatments are indicated by *β* (*p* < 0.05). (B) Colloidal stability assessment of GO and EC@GO (10 mg L^−1^), in ultrapure water (UW) and EPA medium (EPA), measured by the amount of suspended materials over 24 h by UV-vis spectroscopy. (C) Visual inspection of GO and EC@GO dispersions (10 mg L^−1^) over 24 h. (D) Illustration of the amount of suspended materials after 24 h of analysis. (E) Colloidal stability of GO and EC@GO at 10 mg L^−1^ by DLS.

Acute toxicity assays were conducted with young adult organisms to evaluate nematode survival after 24 h of exposure to GO and EC@GO at concentrations ranging from 0.0001 to 10 mg L^−1^. Fig. 3A demonstrates that, at 0.1 mg L^−1^ of GO, nematode survival was reduced to 72 ± 4%, reaching 50 ± 6% at 10 mg L^−1^. In contrast, no adverse effect was observed on the survival of nematodes exposed to EC@GO, as more than 90% of organisms remained alive at all concentrations tested. Therefore, only bare GO impaired nematode survival, indicating that the *E. coli* ecocorona suppressed the adverse effects of GO on nematode survival, at least over the timescale of these experiments.

Few studies have been conducted to identify the acute toxicity of GO to the *C. elegans* model. Wu *et al.* (2013) did not observe lethality of nematodes exposed to concentrations ranging from 0.1 to 100 mg L^−1^.^59^ Similarly, Chatterjee *et al.* (2016) did not report lethality at concentrations varying from 10 to 100 mg L^−1^.^8^ In contrast, Chatterjee *et al.* (2017)^8^ and Li *et al.* (2017)^60^ proved that GO affected the survival of nematodes at concentrations higher than 50 mg L^−1^.

Our data, however, evidence that lower concentrations (*i.e.*, ≥0.1 mg L^−1^) may induce lethality in *C. elegans*, as also confirmed by two previous studies from our group.^6,7^ These contrasting results may be ascribed to differences in the physicochemical properties of the GO utilised, exposure conditions adopted and the colloidal stability of GO in different media, which are composed of monovalent and divalent ions that can affect the colloidal behaviour of NMs over the assay duration.^61^

In our study, moderately hard reconstituted water (EPA medium) was applied to conduct the biological assays, as it is the recommended medium by the EU-funded NanoReg project for testing the toxicity of NMs.^27^ However, a critical point regarding dispersion stability was observed, as GO was not stable for 24 h in EPA. The amount of GO in suspension decreased over time. At the end of the experiment, the total amount of GO was deposited in the bottom of the well. On the other hand, EC@GO exhibited greater stability in the EPA medium; despite some sedimentation, 40% of EC@GO remained suspended after 24 h (Fig. 3B–D).

To further elucidate these differences in dispersion behaviour, DLS measurements were performed (Fig. 3E). In ultrapure water, GO remained relatively stable over time, whereas EC@GO exhibited increased hydrodynamic diameters, indicating aggregation induced by the presence of the biomolecular coating. In EPA medium, both GO and EC@GO showed a marked increase in hydrodynamic diameter, confirming aggregation/agglomeration under higher ionic strength conditions.

DLS measurements showed that both materials formed aggregates with hydrodynamic diameter in EPA medium of up to 2 µm. This size range is compatible with ingestion by *C. elegans*, as the mouth of L3–L4 stage nematodes is approximately 4 µm.^13,62^ Since the organisms used in this study were at the L3–L4 stage, both GO and EC@GO remained physically ingestible under the experimental conditions.

Our results suggest that, although nematodes were exposed to the same nominal concentrations of GO and EC@GO, the effective doses that reached the organisms (which are at the bottom of the wells) differed. Hence, nematodes might have been exposed to higher concentrations of GO compared to EC@GO, which may partly explain why they were more adversely affected by GO. Comparable results regarding this toxicological response were also observed in our earlier study, which demonstrated that a BSA coating improved the colloidal stability of GO, thereby alleviating its toxic effects on nematode survival.^6^

While exposure dose exerts a key role in the toxicological response, the internalized fraction is of significant importance in toxicity, as it is the portion that reaches the target organs and is toxicologically active.^63^ In our study, confocal Raman spectroscopy was exploited to assess the uptake and biodistribution of GO and EC@GO in different tissues and organs of *C. elegans*. GO has characteristic Raman fingerprints (D and G-bands), enabling easy localization inside the worms without using exogenous labels that can come off its surface, leading to potential misinterpretations regarding uptake and localisation.^64^

Depth profiles were drafted from the intensity of characteristic D-bands recorded to confirm the presence of materials inside the nematodes, similar to what was done and explained in our previous work.^6^ The occurrence of asymmetric peaks and the increase of intensity signal after the laser penetrated the cuticle (point zero) were considered indicative that the detected GO signal was not restricted to the external surface of the organism. The persistence and increase of GO-related signal beyond the cuticle, where surface-associated signals are expected to decrease, suggest the presence of GO within the nematode body.

**Fig. 4:**
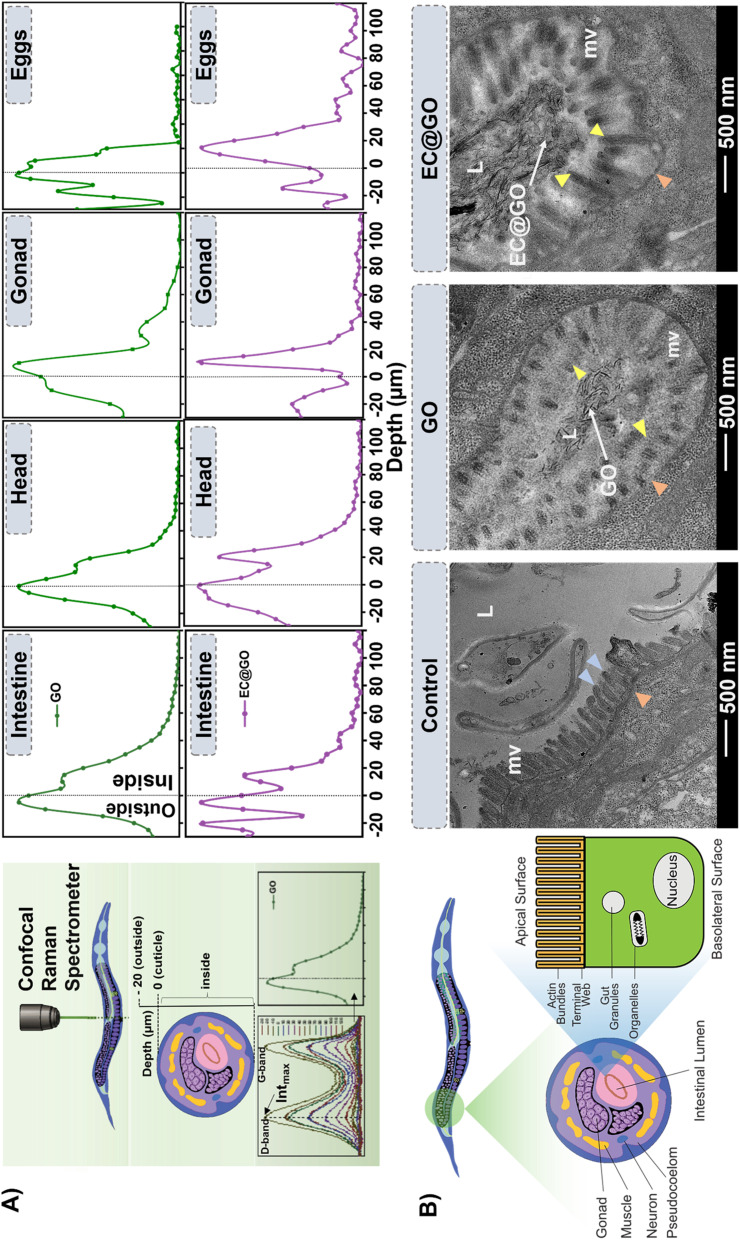
Biodistribution, internalization, and effects of GO on *C. elegans* intestinal barrier. (A) Schematic representation of the study performed using confocal Raman spectroscopy to assess NM internalization, followed by the results of GO and EC@GO (at 10 mg L^−1^) in *C. elegans* tissues analysed by confocal Raman spectroscopy from −25 to 120 µm depth (negative values = outside the nematode; positive values = inside the nematode). Point zero represents the nematode cuticle. (B) Schematic representation of the ultrastructural level analysis of the intestinal barrier performed by transmission electron microscopy (TEM), along with representative transmission-electron micrographs of non-exposed *C. elegans* (control) and nematodes treated with GO and EC@GO at 10 mg L^−1^. Microvilli and lumen are represented by mv and L, respectively. Orange arrowheads indicate the terminal web. Blue arrowheads evidence the presence of healthy microvilli, whereas yellow arrowheads indicate intestinal damage of microvilli.

Fig. 4A reveals the occurrence of GO and EC@GO in the nematode's intestine, head, gonad, and spermatheca-associated regions, suggesting that both materials were internalized despite their aggregated state during the biological experiments. These observations were supported by the depth-resolved Raman profiles, which show asymmetric peaks and an increase of intensity signal within the nematode, after penetrating the cuticle, indicating that the detected signal was not restricted to the external surface of the organism. In addition to the Raman analyses, dark aggregates were visually observed in the intestinal lumen of exposed nematodes (Fig. S4), providing complementary evidence of material accumulation within the digestive tract. While these findings support the internalization of GO and EC@GO, the exact anatomical localization of the material was interpreted with caution due to the limitations of Raman depth profiling and the potential morphological changes associated with fixation procedure. For reference, Raman spectra of the materials were provided in the SI (Fig. S5), and representative spectra acquired from unexposed and exposed organisms are shown (Fig. S6).

The alimentary system of *C. elegans* includes the mouth, pharynx, intestine, rectum, and anus. By activating sensory neurons, *C. elegans* captures food (bacteria), which is broken down by the pharyngeal grinder and transported to the intestine. Together with food, nematodes can actively ingest NMs, which may cross their intestines, disrupting their biological function and leading to the material's translocation to other tissues.^10,12,65^

Since the internalization of GO inside the nematode body can impact its intestinal barrier, its tissue was evaluated at the ultrastructural level by transmission electron microscopy (TEM). Fig. 4B shows the microvilli of the control group with typical morphology, well-defined invaginations and actin-rich microfilaments that form the apical membrane and are anchored in the cytoskeletal structure denominated as the terminal web. TEM images of NM exposed organisms reveal the accumulation of GO and EC@GO within the nematode lumen, but do not allow direct visualization of trans-epithelial transport or precise subcellular localization of the material. This accumulation led to damage in the apical membrane surface in both treatments, evidenced by interruptions in the actin microfilaments. However, in organisms exposed to EC@GO, the actin microfilaments appeared denser and more continuous than those exposed to GO, suggesting that EC@GO caused less structural damage to the nematode microvilli. Within the resolution and scope of the TEM analysis, no definitive evidence of GO localization within lipid droplets or other subcellular compartments was observed.

Based on the confocal Raman spectroscopy and TEM results, GO and EC@GO affected the *C. elegans* intestine and were detected in regions beyond the intestinal lumen. Similar results for GO were obtained by Wu *et al.* (2013), who also identified disruption of microvilli structure, increased permeability of the intestinal barrier and distribution of GO to surrounding mitochondria in intestinal cells.^59^

Our results suggest that the intestines of nematodes exposed to EC@GO were less affected than those exposed to GO. In a previous study, we found that, although GO had increased the permeability of the *C. elegans* intestinal barrier, this effect was attenuated by a bovine serum albumin (BSA) coating.^6^ A similar protective effect of the biocorona was also reported by Gonzalez-Moragas *et al.* (2015), who demonstrated that BSA prevented direct contact between superparamagnetic iron oxide nanoparticles and the *C. elegans* intestinal environment, thereby alleviating their adverse effects.^66^ This suggests that the *E. coli* ecocorona could have hindered direct interactions between GO and the *C. elegans* intestine, leading to a less severe impact on its microvilli.

Due to the evidence that both GO and EC@GO were internalized by the nematodes, we investigated whether exposure to the materials affected the neurons and physiological function of *C. elegans* gonads. Two transgenic *C. elegans* strains (MD701 and AML175) were exploited in our experiments. In AML175 strain (*wtfIs3 [rab-3p::NLS::GFP + rab-3p::NLS::tagRFP*]), neurons are marked with nuclear-localized GFP and tagRFP that fluoresce in living cells. Thus, a decrease in total fluorescence intensity is indicative of neuronal damage. In the MD701 strain [(*bcls39 [lim-7p::ced-1::gfp + lin-15(+)*], the cell surface phagocytic receptor CED-1 is labelled with green fluorescence proteins (CED-1:GFP) and is expressed around apoptotic germline cells. Therefore, the MD701 strain is a valuable tool for tracking the impairment of the *C. elegans* reproductive system by pollutants.

By exposing the AML175 *C. elegans* strain to 0.0001 to 10 mg L^−1^ of GO and EC@GO, a decrease in fluorescence intensity of the neural system marked with GFP and tagRFP was observed in nematodes exposed to 10 mg L^−1^ of GO (92.7 ± 1.4%). In contrast, the fluorescence intensity of worms exposed to EC@GO (98.4 ± 1.7%) was similar to that of the controls (100 ± 1.9%) (Fig. S7).

The nervous system is the most complex organ of *C. elegans* because it controls all essential functions of the nematode, including feeding, development, movement, metabolism, and reproduction.^9^ When nematodes are exposed to NMs, the nervous system is a primary target organ due to its sensitivity and central role in regulating vital functions.

Our findings suggest that GO induced neuronal damage in *C. elegans*. Similar results were reported by Kim *et al.* (2020), who utilised confocal Raman spectroscopy and observed GO deposition in the head region of *C. elegans*.^9^ This was accompanied by significant alterations in locomotor behaviour, such as changes in stop time and speed. The volume of neurotransmitters in dopaminergic and glutamatergic neurons was reduced, as well as the expression of genes required for the function of AFD thermosensory neurons, confirming the potential neurotoxic effect of GO. To date, no studies have addressed the influence of the *E. coli* ecocorona on GO's neurotoxic effects. However, our results suggest that only GO affected the functionality of *C. elegans* neurons, with no impact form the EC@GO exposure. Further research is required to elucidate the mechanisms by which the ecocorona formation suppresses GO neurotoxicity.

**Fig. 5:**
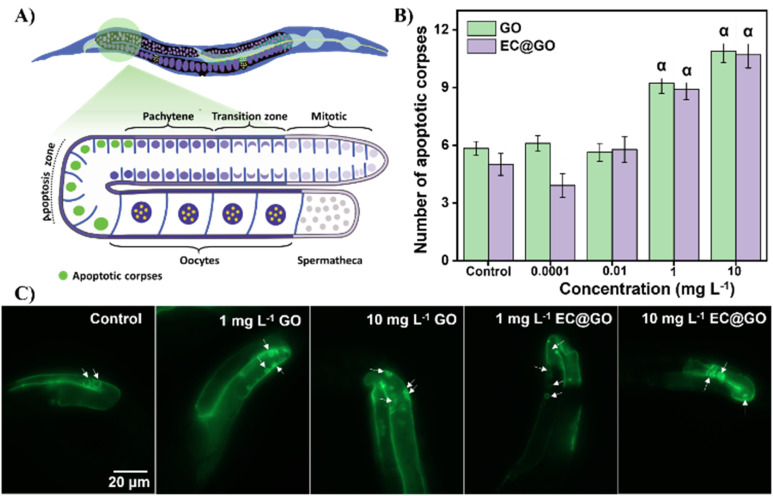
Assessment of germline apoptosis induction in *C. elegans* by using the MD701 strain [(*bcls39 [lim-7p::ced-1::gfp + lin-15(+)*)]. (A) Schematic diagram of germline apoptosis in *C. elegans*. (B) Effect of GO and EC@GO on the induction of apoptotic corpses in the germline of *C. elegans*. (C) Representative fluorescence images of apoptotic induction in the MD701 strain. White arrows highlight apoptotic cells, and α indicates significant differences between control and treatments (*p* < 0.05), with no statistically significant difference between treatments.

Fig. 5 shows the effect of GO and EC@GO on the induction of germline apoptosis in *C. elegans*. In non-exposed nematodes, the expression of CED-1::GFP clusters around germline cells was 5.8 ± 0.4. A significant increase in the expression of CED-1::GFP clusters was observed for nematodes exposed to 1 and 10 mg L^−1^ of GO (9.2 ± 0.5 and 10.9 ± 0.6, respectively) and to the same concentrations of EC@GO (8.9 ± 0.5 and 10.7 ± 0.7, respectively) (Fig. 5B). However, the magnitude of this rise (∼1.5–2.0 fold) is not statistically different between materials, suggesting that GO and EC@GO affected the nematode germline cells similarly, with limited to no reduction of the effect due to ecocorona formation.

In a young nematode, the germline mitotic region contains about 100 self-renewing cells that produce differentiating gametes. Apoptosis, a programmed event, plays a crucial role in oogenesis (the differentiation of the ovum) by removing defective cells, maintaining genomic stability and regulating sperm production.^67^ However, environmental stressors can disrupt this process, leading to a reduced number of mature oocytes and compromised offspring development, as has been observed in nematodes exposed to GO.^68,69^ Studies have reported suppression of spermatogenesis and offspring production when GO reaches the *C. elegans* gonad.^70^ In addition, GO induces apoptosis corpses, cell cycle arrest and DNA fragmentation, affecting the nematode reproduction.^71^

In our study, and EC@GO were detected in gonad and spermatheca-associated regions, coinciding with increased germline apoptosis. Although the *E. coli* ecocorona could have hindered direct interactions between GO and the *C. elegans* intestine, alleviating the effects of GO on the microvilli, this coating may also have enhanced the biocompatibility of GO within the nematode gut. Such behavior could contribute to the persistence of GO-associated effects in reproductive tissues, which may explain why the *E. coli* ecocorona did not mitigate the apoptotic effect induced by GO. Raman signals were also detected in spermatheca-associated regions; however, the precise localization of the material within reproductive structures and the route by which it reached these regions remain to be further investigated. In brief, our findings reveal that GO was internalized and distributed throughout different regions of the *C. elegans* body, impacting the microvilli of the intestinal barrier. GO was toxic for nematodes, reducing survival, and damaging germline cells and neurons. The *E. coli* ecocorona-coated GO was also internalized and detected in regions beyond the intestinal lumen, where it impaired the germline cells. However, nematodes exposed to this coated material did not die, and their neurons were preserved. The microvilli of nematodes exposed to EC@GO were less affected than those exposed to GO. Therefore, these results demonstrate that the *E. coli* ecocorona has a positive effect in protecting the nematodes against the adverse effects of GO in single exposure scenarios at least over acute timescales.

### 3.3. *E. coli* ecocorona aggravates the combined toxicity of GO with silver ions

After investigating whether the *E. coli* ecocorona alters GO toxicity, we selected a metallic co-pollutant model (*i.e.*, silver ions) to be combined with GO. In this experimental set, a fixed nontoxic concentration of GO or EC@GO (0.1 µg L^−1^) was applied in combination with increasing concentrations of silver ions. The aim was to assess whether the ecocorona influences the effects of other pollutants in co-exposure scenarios.

**Fig. 6:**
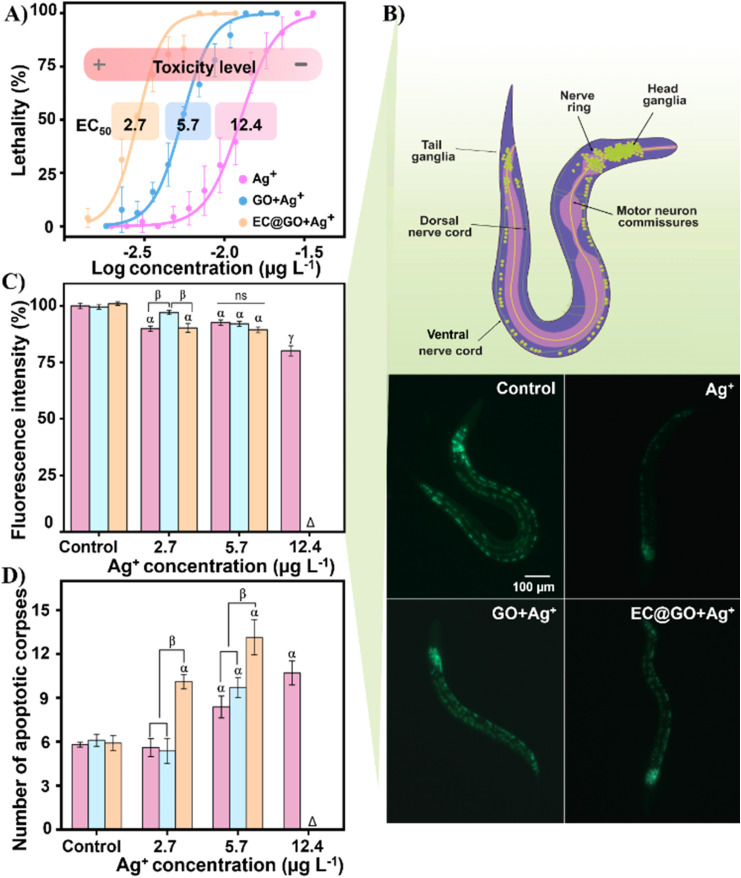
*E. coli* ecocorona effect on combined toxicity of GO and EC@GO (at 0.1 µg L^−1^) with different Ag^+^ concentrations. (A) Dose–response curves (with the *x*-axis representing the applied varying silver dose) and respective LC_50_ values, (B) schematic illustration of the AM175 *C. elegans* strain, in which neurons are marked with nuclear-localized GFP and tagRFP that fluoresce in living cells. (C) Neuronal damage indicated by the relative fluorescence intensity of neurons and (D) apoptosis on germline cells, indicated by the number of apoptotic corpses. Data are present as mean ± SEM. Symbols: *α* indicates the treatments differing from the control; *β e γ* suggests differences between treatments; *Δ* represents data not applicable because of no nematodes surviving at this concentration (*p* < 0.05).

Fig. 6A shows the dose–response curves for single and combined exposure, as well as the respective LC_50_ values, which represent the concentration that is lethal for 50% of the exposed organisms. The LC_50_ for nematodes exposed to Ag^+^ was 12.4 µg L^−1^ (95% CI = 11.8–13.1), whereas the LC_50_ for nematodes exposed to Ag^+^ combined with GO (GO + Ag^+^) was 5.7 µg L^−1^ (95% CI = 5.4–6.0) and 2.7 µg L^−1^ (95% CI = 2.5–2.9) for nematodes exposed to *E. coli* ecocorona-coated GO with Ag^+^ (EC@GO + Ag^+^). Therefore, the LC_50_ decreased in the co-exposure scenarios, indicating an enhancement in toxicity due to the combination of Ag^+^ with both materials, with the impact of treatments on nematode survival following the order: EC@GO + Ag^+^ > GO + Ag^+^ > Ag^+^.

Fig. 6B illustrates the AML175 *C. elegans* strain, applied as a platform to investigate the neuronal effects promoted by the co-exposure scenarios. Fig. 6C then presents the neuronal impacts of single and combined exposure. Our data reveals a decrease in GFP fluorescence intensity in nematodes exposed to 2.7 µg L^−1^ of Ag^+^ (alone or combined with 0.1 µg L^−1^ of EC@GO), reducing from 100 ± 1% (control) to 90 ± 1% and 90 ± 2%, respectively. At 5.7 µg L^−1^ Ag^+^ (alone or combined with 0.1 µg L^−1^ of GO or EC@GO), all treatments induced a similar reduction in fluorescence intensity as observed at 2.7 µg L^− 1^ Ag^+^. Notably, nematodes exposed to 12.4 µg L^−1^ of Ag^+^ showed a significant decrease in GFP intensity, reducing to 80 ± 2% of the control value. These data suggest that single and combined exposure led to neuronal alterations, with Ag^+^ and EC@GO + Ag^+^ showing the highest impact at lower concentrations.

The effects of the single and combined exposure were also evaluated on the *C. elegans* germline. Fig. 6D shows 5.8 ± 0.2 apoptotic corpses in unexposed nematodes (control). An increase in the occurrence of apoptosis was observed in nematodes exposed to EC@GO + Ag^+^ in which the Ag^+^ concentration was 2.7 µg L^−1^, which exhibited 10.1 ± 0.5 apoptotic corpses, whereas Ag^+^ and GO + Ag^+^ did not affect the nematodes at this concentration. At 5.7 µg L^−1^ Ag^+^, EC@GO + Ag^+^ was the most apoptotic treatment, with 13.1 ± 1.2 apoptotic corpses observed, while Ag^+^ alone and GO + Ag^+^ showed similar number of apoptotic corpses, 8.3 ± 0.7 and 9.7 ± 0.7, respectively. Nematodes exposed to 12.4 µg L^−1^ of Ag^+^ exhibited 10.7 ± 0.8 germ cell corpses, but this concentration could not be tested for EC@GO + Ag^+^ and GO + Ag^+^ because no nematodes survived. These data indicate that EC@GO + Ag^+^ was the worst treatment for the *C. elegans* germline, as it induces apoptosis at lower concentrations than GO + Ag^+^ and Ag^+^. Considering the diversity of Ag^+^ binding proteins this is not overly surprising.^72,73^ However, the magnitude of the impact is a surprise, particularly under co-exposure conditions with EC@GO. Although silver is widely used and continuously released into in the environment as residues from the photography, tobacco and other industrial activites.^74^ Reported levels of silver in natural water are generally in the ng L^−1^ range, occasionally reaching low µg L^−1^ levels.^74^ In the present study, toxic effects were observed in the low µg L^−1^ range, indicating that biologically relevant responses may occur near the upper bounds of environmentally reported levels.

Previous studies have reported that some heavy metals, including iron,^75^ lead and mercury,^76^ copper, zinc, cadmium, chromium,^77^ and nickel,^78^ can be toxic to the survival of *C. elegans*. In agreement with our results, Starnes *et al.* (2015) described an LC_50_ of 7.5 µg L^−1^ (95% CI = 7.01–7.89) for nematodes exposed to Ag^+^ in moderated hard reconstituted water.^79^ Besides the effects on survival, other studies have identified heavy metals as inducers of germline apoptosis and neurotoxic damage in *C. elegans*.^80,81^ Exposure to Ag^+^ has been associated with declining offspring numbers and reproductive rate.^79,82^ Silver ions have been found to disrupt the morphology and development of AFD sensory neurons, which are crucial for worm perception behaviour.^76^ A reduction of nematode body bends and impaired learning performance has been noticed, indicating potential dysfunction in the nervous system.^83^ This previous knowledge supports the idea that Ag^+^ may have disrupted the functionality of *C. elegans* reproductive and neuronal system, as it induced the death of germline cells and affected the neurons.

The co-exposure of GO or EC@GO with Ag^+^ has never been evaluated using the *C. elegans* model. It is evident in our results that GO and EC@GO worsened the toxicity of silver, as the co-exposure of GO with Ag^+^ aggravated silver lethality by 210%, and the combination of EC@GO with Ag^+^ increased silver lethality by 460%. Likewise, EC@GO also worsened the degree of apoptosis induced by Ag^+^ in the germline.

Wang *et al.* (2019) reported that TiO_2_ NPs combined with Cd^2+^ led to the translocation of these ions to nematode gonads and embryos, resulting in germline apoptosis.^26^ Similarly, in our study, we applied confocal Raman spectroscopy to investigate the biodistribution of GO and EC@GO in nematodes exposed to GO + Ag^+^ and EC@GO + Ag^+^.

**Fig. 7:**
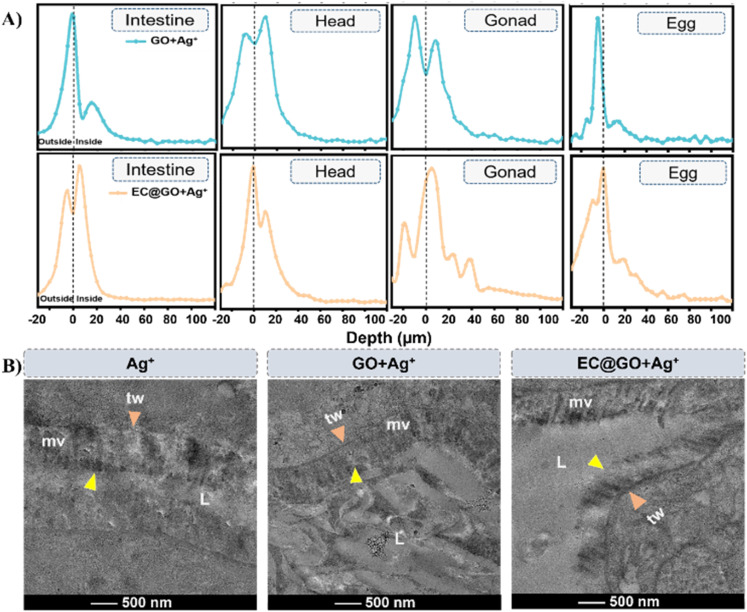
Biodistribution, internalization and effects of GO + Ag^+^ and EC@GO + Ag^+^ on *C. elegans* intestinal barrier. (A) Internalization of GO + Ag^+^ and EC@GO + Ag^+^ in *C. elegans* tissues analyzed by confocal Raman spectroscopy from −25 to 120 µm depth; point zero represents the nematode cuticle. (B) Transmission-electron micrographs of nematodes exposed to Ag^+^, GO + Ag^+^ and EC@GO + Ag^+^ at the LC_50_ values. Microvilli and lumen are represented by mv and L, respectively. Orange arrowheads indicate the terminal web. Yellow arrowheads indicate intestinal damage specifically of microvilli.

Fig. 7A shows GO-related Raman signals in multiple anatomical regions of *C. elegans*, including intestine, head, gonads, and spermatheca-associated regions), supporting the internalization of both materials under co-exposure conditions. It is important to note that Raman spectroscopy enabled the detection of GO/EC@GO through its characteristic D and G bands but did not allow the direct detection of Ag.

TEM was employed to assess the ultrastructural alterations promoted by Ag^+^, GO + Ag^+^ and EC@GO + Ag^+^ in the *C. elegans* intestine. Exposure to Ag^+^ resulted in a significant loss of microvilli, characterized by the disintegration of actin filament bundles, disruption of the apical membrane, and a loss of integrity in the terminal web (Fig. 7B). Among the treatments, Ag^+^ exposure induced the most pronounced physiological damage. However, harmful effects were noted in nematodes exposed to GO + Ag^+^ and EC@GO + Ag^+^, with a highly disordered intestinal brush border. In addition, nematodes exposed to EC@GO + Ag^+^ exhibited a pronounced discontinuity effect, whereby the apical membrane became irregular and fragmented, indicating that EC@GO + Ag^+^ has a stronger affinity for the microvilli surface compared to GO + Ag^+^.

In summary, our data reveal that Ag^+^ exposure significantly compromised survival, neuronal integrity, germline cells and intestinal barrier of *C. elegans*. The co-exposure of Ag^+^ with bare and ecocorona-coated GO is also a concern, as silver lethality increased by 210% when it was jointly exposed with bare GO and by 460% when combined with *E. coli* ecocorona-coated GO. Besides its worst effect on nematode survival, EC@GO + Ag^+^ showed a strong affinity for the microvilli surface and was the most damaging treatment to the germline physiological function. Therefore, the behaviour/effect of EC@GO + Ag^+^ will be further discussed to elucidate why the *E. coli* ecocorona increases Ag^+^ toxicity to a higher degree than bare GO.

### 3.4. Revealing the contribution of *E. coli* ecocorona in aggravating metal ion toxicity

Several authors have reported nanomaterials as carrier agents of pollutants.^25^ This process, often referred to as the “Trojan horse effect”, occurs when nanomaterials adsorb contaminants, thereby enhancing the uptake and transport of these pollutants into organisms.^52^ This enhanced uptake can lead to an increase in toxicity.^25^

**Fig. 8:**
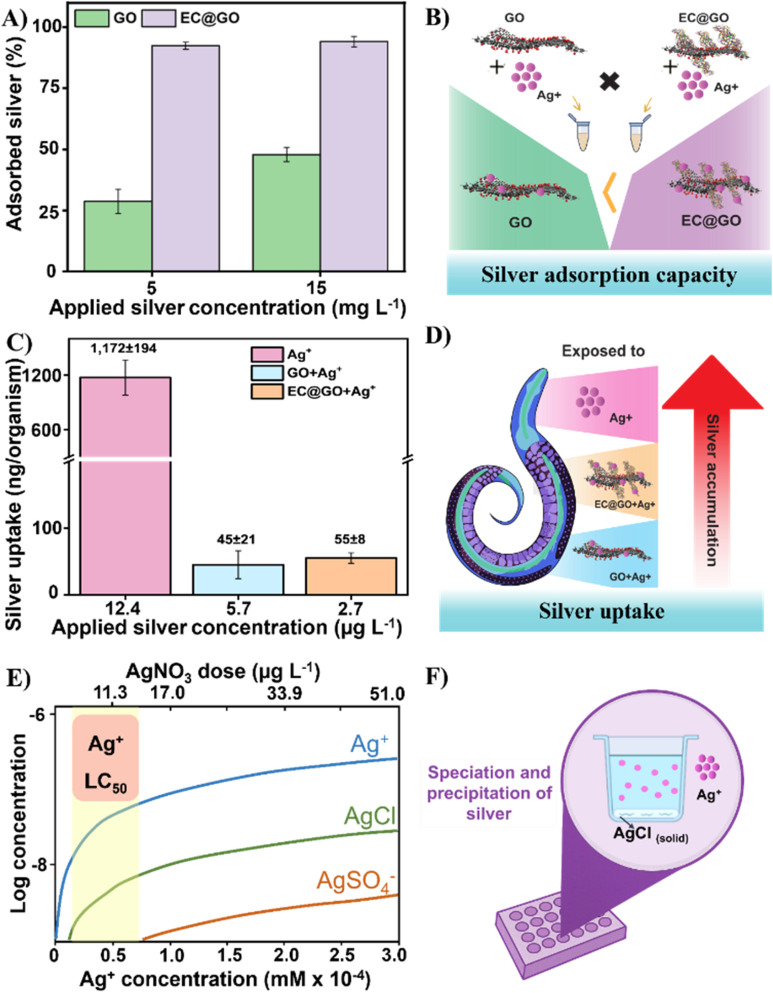
Demonstration elucidating why the *E. coli* ecocorona aggravated the silver toxicity. (A) Total adsorbed silver (%) by GO and EC@GO Data are present as mean ± SEM. (B) Diagram illustrating the higher capacity of EC@GO to adsorb Ag than GO. (C) Total silver concentration internalized by nematodes, which were digested and subsequently analyzed by ICP-MS. Data are present as mean ± SEM. (D) Diagram illustrating the level of silver accumulation by the nematodes exposed to Ag^+^, GO + Ag^+^ and EC@GO + Ag^+^. Considering the initial concentrations at which organisms were exposed (*i.e.*, LC_50_ values). (E) Predicted silver speciation calculated using Spana® software under the experimental conditions (*i.e.*, EPA medium and silver concentration). The shaded region highlights the Ag^+^ LC_50_ concentration range used for biological interpretation. Within this concentration range, Ag^+^ and AgCl were the predominant silver species predicted by the model. (F) Schematic representation of the predominant silver species expected to be present in EPA medium during Ag^+^ exposure, illustrating the coexistence of dissolved Ag^+^ and precipitated AgCl under the experimental conditions.

According to our biological results, Ag^+^ lethality was enhanced due to its interaction with bare and coated GO. To further explore this, we decided to investigate the capacity of GO to adsorb Ag by applying a fixed concentration of GO (10 mg L^−1^) and two different Ag^+^ concentrations (5 and 15 mg L^−1^). Our results demonstrate that bare GO adsorbed 29 ± 2% and 48 ± 3% of Ag, respectively, while *E. coli* ecocorona-coated GO retained 92 ± 2% and 94 ± 2% of the total Ag (Fig. 8A). The adsorption capacity of GO to remove 5 and 15 mg L^−1^ of Ag^+^ was 2.8 ± 0.2 mg of Ag per g of GO and 4.8 ± 0.7 mg of Ag per g of GO, respectively. While the ecocorona-coated GO demonstrated a remarkable capacity, adsorbing 5.5 ± 0.1 mg of Ag per g of EC@GO and 15.7 ± 0.1 mg of Ag per g of EC@GO, for 5 and 15 mg L^−1^ Ag^+^ applied, respectively. It is noteworthy, therefore, that the *E. coli* ecocorona-coated GO adsorbed a significantly a higher concentration of silver than bare GO (Fig. 8B), likely driven by the high affinity of many proteins for Ag^+^.^72,84^

GO has a strong adsorption capacity for heavy metals due to oxygenated functional groups on its surface (carboxyl, hydroxyl and epoxy groups) that work as binding sites for metal ions.^52^ Additionally, functional groups from proteins, including –SH, –NH and –COOH, are also highly effective in adsorbing those components.^85^ Our XPS and FTIR data revealed an increase in the diversity of functional groups on the GO surface after ecocorona formation. Then, those functional groups could have been responsible for the high Ag adsorption capacity of EC@GO.

Further investigations were performed to assess whether the concentration of Ag adsorbed onto EC@GO could have influenced the internalization and accumulation of Ag by *C. elegans*. Nematodes were exposed to the LC_50_ values established in our single and co-exposure assays: Ag^+^ (12.4 µg L^−1^), GO + Ag^+^ (5.7 µg L^−1^), and EC@GO + Ag^+^ (2.7 µg L^−1^). The total Ag concentration internalized by organisms was then quantitatively analyzed using ICP-MS.

Nematodes exposed to Ag^+^, GO + Ag^+^ and EC@GO + Ag^+^ accumulated 1172 ± 194, 45 ± 21, and 55 ± 8 ng of Ag per organism, respectively (Fig. 8C). In contrast, the background concentration of Ag in unexposed worms was 1.1 ± 0.7 ng per organism. Considering the different initial concentrations of silver to which organisms were exposed, it was found that each nematode accumulated 9.5 ± 1.6%, 0.8 ± 0.3%, and 2.1 ± 0.3% of the available silver when exposed to Ag^+^, GO + Ag^+^ and EC@GO + Ag^+^, respectively (Fig. 8D). Therefore, the amount of Ag accumulated by the nematodes followed this sequence: Ag^+^ > EC@GO + Ag^+^ > GO + Ag^+^.

Organisms exposed to EC@GO + Ag^+^ accumulated twice as much Ag as those exposed to GO + Ag^+^, evidencing that the ecocorona coating significantly enhanced Ag bioaccumulation by the nematodes in the co-exposure mode. This effect is likely due to the higher dose of Ag delivered by EC@GO + Ag^+^ to the nematodes during the biological assays, as EC@GO adsorbs around 300% more silver than GO. Consequently, this contributed to EC@GO + Ag^+^ being more toxic than the other treatments. We cannot exclude the possibility that *E. coli* ecocorona coating could have made the EC@GO + Ag^+^ complex more biocompatible to *C. elegans* because *E. coli* is its bacterial food source, increasing Ag assimilation. As EC@GO + Ag^+^ has Ag on its surface, this metal could have been desorbed from the EC@GO surface, being released during its internalization within the nematode tissues, justifying why EC@GO + Ag^+^ promoted the discontinuity of the apical membrane and was the worst treatment for the nematode germline. All those factors demonstrate that the *E. coli* ecocorona may have acted as a Trojan horse, enhancing the uptake and transport of silver within *C. elegans* and releasing the Ag^+^ in areas where it might not reach alone. It is possible that proteins in the various *C. elegans* tissues have higher affinity for the Ag^+^ than the *E. coli* proteins in the ecocorona, a possibility that we will explore in subsequent work.

The lethality of Ag^+^ was also increased due to its interaction with bare GO. Therefore, GO may have also acted as a carrier agent for Ag, promoting its internalization and biodistribution within the nematode. However, the effects of GO + Ag^+^ were less notable than EC@GO + Ag^+^ and were similar to those observed in the direct Ag^+^ exposure in the *C. elegans* secondary organs. This outcome may be attributed to the fact that the bare GO does not have its surface coated by *E. coli* biomolecules, which could make it less recognizable for uptake, and influencing the silver desorption in the tissues. In addition, GO has a lower amount of Ag on its surface, resulting in a lower concentration of Ag internalized by nematodes exposed to GO + Ag^+^ in comparison to EC@GO + Ag^+^. Therefore, although GO also acted as a silver carrier, it caused less harmful effects than EC@GO combined with Ag^+^.

The colloidal stability of GO with heavy metals during the toxicity assays is a critical factor that cannot be overlooked. We investigated the behaviour of GO + Ag^+^ and EC@GO + Ag^+^ in EPA medium over 24 h (Fig. S8-A). Our data suggest that after 6 h of exposure, the percentage of suspended GO + Ag^+^ and EC@GO + Ag^+^ decreased to 45.4 ± 4.6% and 35.3 ± 3.1%, respectively. By the end of the experiment (24 h), only 4.6 ± 2.5% and 20.1 ± 1.3% of GO + Ag^+^ and EC@GO + Ag^+^ respectively remained suspended (Fig. S8-B and S8-C).

DLS measurements demonstrated that both GO + Ag^+^ and EC@GO + Ag^+^ agglomerated/aggregated during the assays, reaching hydrodynamic diameters around 2 µm over time (Fig. S8-D).

Wang *et al.* (2018) reported that interactions between heavy metals (such as cadmium, arsenic or nickel) and TiO_2_ nanoparticles reduced the surface energy of the NMs, leading to agglomeration and aggregation events. This, in turn, alters the vertical distribution of these pollutants causing them to settle to the bottom of the exposure vessel, and thus resulting in prolonged exposure of *C. elegans* to heavy metals.^26^

Similarly, in our study, the low colloidal stability of GO + Ag^+^ and EC@GO + Ag^+^ may have affected their proximity and availability to nematodes, thereby influencing both the uptake and toxicity of Ag^+^. However, despite aggregation, the hydrodynamic diameters remained within a size range compatible with ingestion by nematodes, indicating that both suspended and sedimented fractions were biologically available.

Moreover, although organisms directly exposed to Ag^+^ presented the highest internalized Ag content, Ag^+^ treatment was the least toxic to *C. elegans* survival. Silver toxicity is known to depend on silver speciation, which is modulated by the media composition.^80^ As such, to better understand our results, we modelled the speciation of Ag expected in the EPA medium, considering the conditions of our experiments. At 12.4 µg L^−1^ (*i.e.*, LC_50_ for Ag^+^ exposure), two silver species were found: Ag^+^ (mostly) and AgCl (solid) (Fig. 8E), suggesting that these two species likely impacted Ag toxicity. Since AgCl is a precipitated form of Ag, nematodes may have ingested a high amount of Ag through contact with the sediment at the bottom of nematodes exposed to Ag^+^. However, according to the literature, AgCl is less toxic than Ag^+^ to organisms.^81^ This may explain why nematodes internalized a high amount of silver in the direct exposure but exhibited less severe effects. While this interpretation refers to the direct Ag + exposure, differences in silver speciation may also have contributed to the toxicity observed under GO + Ag^+^ and EC@GO + Ag^+^ exposure conditions.

Additionally, it is possible that the metallothioneins played a significant role in modulating Ag toxicity, particularly *mtl-1* and *mtl-2*, which have a high affinity for metals such as Hg^2+^, Cu^+^, Cd^2+^, Zn^2+^, and Ag^+^.^80,86^ These proteins hinder the diffusion of heavy metals within cells, preventing their binding in vital enzymes and proteins responsible for maintaining biological functions in organisms.^87^ Therefore, we assumed that metallothioneins could have alleviated the toxic effects of Ag^+^ on the nematodes.

Briefly, our findings demonstrate that the effects of Ag^+^ were modulated by its speciation in the EPA medium and potentially by metallothioneins, which may have limited its cellular diffusion. Bare GO may have acted as a silver carrier agent; however, it induced less harmful effects than EC@GO + Ag^+^, which enhanced GO's adsorption capacity and promoted higher Ag internalization. Furthermore, the *E. coli* ecocorona may have facilitated silver desorption on the microvilli and gonads of *C. elegans*, causing adverse effects on both tissues. These outcomes emphasize the critical role of the *E. coli* ecocorona in increasing the GO's Trojan horse effect.

### 3.5. Implications of the *E. coli* ecocorona for nanosafety research

Our study provides the first evidence that, despite the *E. coli* ecocorona mitigating GO toxicity, it may also act as a Trojan horse by enhancing silver toxicity. This dual effect demonstrates that interactions between nanomaterials and environmentally acquired biomolecular coatings can substantially alter nanomaterial behaviour and biological outcomes. In the present study, the interaction of GO with *E. coli* biomolecules modified its surface properties, reduced its intrinsic toxicity, and simultaneously enhanced its capacity to adsorb and transport silver ions. These findings reinforce the importance of considering ecocorona formation when assessing nanomaterial safety and environmental behaviour.

It is important to note that the ecocorona investigated in this study was generated from intracellular biomolecules released from *E. coli*, representing a simplified and controlled model system. While this approach enables the investigation of defined biomolecular interactions, it does not fully capture the complexity of *in vivo* conditions, where nanomaterials interact dynamically with intact microorganisms, extracellular polymeric substances (EPS), metabolites, and organism-derived biomolecules. In addition, although proteomic analysis enabled the identification of proteins associated with the ecocorona, other biomolecular components potentially present in the system (*e.g.*, lipids, nucleic acids, and small metabolites) were not specifically characterized. Therefore, the present findings should be interpreted within the context of a simplified biomolecular corona model, which nonetheless provides relevant mechanistic insights into nanosafety.

Neglecting the ecocorona evaluation not only heightens the risks of inconsistent findings across studies but also hinders the comparability of toxicity data and creates critical gaps in understanding NM behaviour in complex biological environments where the presence of co-pollutants is inevitable. Moreover, it raises significant concerns regarding the relevance and translational applicability of such findings, particularly in environmental and biomedical nanotoxicology contexts.

Therefore, we strongly advocate for the inclusion of ecocorona evaluation as a prerequisite in standard ecotoxicology protocols. Specifically, we propose its integration into the ISO 10872:2020 protocol,^15^ originally designed for testing sediment and soil samples with *C. elegans*, but whose applicability should be analysed and adapted for use with NMs, microplastics and particle-containing mixtures. This approach would enhance comparability between studies and minimize experimental artifacts, thereby supporting the application of nanoinformatic approaches.

Future studies should expand this framework to include the evaluation of *in situ* ecocorona evolution and to distinguish between intracellular and extracellular (*e.g.*, EPS-derived) contributions during exposure and gut passage, as well as to assess the role of different biomolecular classes in ecocorona formation and function. Incorporating such assessments would provide a more comprehensive understanding of NM behaviour in biological systems, ultimately improving the reliability and applicability of nanosafety research for regulatory decision-making and the development of safer nanotechnology applications.

## 4. Conclusions

In this study, we demonstrated for the first time the formation of an *E. coli* protein ecocorona on graphene oxide. This coating significantly altered the GO's physicochemical properties by increasing its roughness and thickness and modifying its surface elemental composition introducing new functional groups. A selective protein binding on GO was observed, with an enrichment of low molecular weight *E. coli* proteins in the acquired ecocorona.

The *E. coli* ecocorona was crucial in mitigating GO toxicity to the *C. elegans* model by suppressing its lethality, preserving its neurons, and reducing damage to the germline and intestinal barrier. On the other hand, the *E. coli* ecocorona increased silver lethality by 460% compared to the direct exposure to Ag^+^ alone, in part due to the speciation of the Ag in EPA medium to the less bioavailable AgCl.

The presence of *E. coli* ecocorona enhanced the capacity of GO to adsorb silver ions by around 300%, which led to an increase in the amount of silver internalized by the nematodes. In addition, the biomolecules from the *E. coli* ecocorona may have made GO combined with silver ions more assimilable by nematodes, which may have promoted the desorption of Ag^+^ in its sensitive tissues at concentrations that would not be possible without the EC@GO carrier or Trojan horse effect. Consequently, adverse effects on *C. elegans* microvilli, neurons and reproductive organs were observed by the exposure of worms to ecocorona-coated GO combined with Ag^+^.

Taken together, our findings demonstrate that although the *E. coli* ecocorona mitigated the adverse effects of GO alone, it acted as a Trojan horse by enhancing Ag^+^ toxicity. Our outcomes not only provide valuable insights into the formation and implications of the *E. coli* ecocorona in nanosafety studies but also highlight its dual effect in modulating graphene oxide toxicity. This perspective emphasizes the importance of the *C. elegans* research community recognizing the need to characterize the *E. coli* ecocorona, or as a mimulus check for ecocorona formation, as a prerequisite in nanosafety assessments. This approach will enable the generation of reliable and representative results, which will enhance data comparability, advance the application of nanoinformatics, improve regulatory decision-making, and promote the development of safe nanomaterials.

## Author contributions

Conceptualization, F. C. and D. S. T. M.; methodology F. ·C.; M. S; F. D.; C.·H. A-J.; investigation, F.·C.; C.·H. A-J.; F.·S. D.; B.·S.·B.; validation, M. S.; R. V.·P.; I. L.; data curation, F.·C.; writing – original draft preparation, F.·C.; writing-review and editing, M. S.; D. S. A.; B.·S.·B.; I. L.; D. S. T. M; supervision, D. S. T. M.; C.·H. A-J.; project administration, F.·C.; funding acquisition, I. L.; D. S. T. M. All the authors have read and agreed to the published version of the manuscript.

## Conflicts of interest

The authors declare the following competing financial interest: F. C. and D. S. T. M. are inventors of the Brazilian Patent BR 10 2022 012202-4, titled “Corona de proteínas carreadora de agentes antinematoides e seu processo de produção” filed on June 20, 2022. This patent details the methodology for coating graphene oxide with *E. coli* biomolecules.

## Supplementary Material

NA-OLF-D6NA00311G-s001

NA-OLF-D6NA00311G-s002

## Data Availability

The data that support the findings of this study are available in the electronic supplementary information (SI). Supplementary information: detailed experimental methods, supplementary figures and tables, additional characterization data, complete proteomic datasets, and supporting experimental results. See DOI: https://doi.org/10.1039/d6na00311g.
