# Supplementary material for: Caenorhabditis elegans in nanosafety research: dual effect of Escherichia coli ecocorona formation on single and combined toxicity of graphene oxide
Source: Nanoscale Adv. 2026 Aug 6;8(19):5535–56. doi: 10.1039/d6na00311g (PMC13444697; doi:10.1039/d6na00311g)
Supplement: Supplementary file 1 [file NA-008-D6NA00311G-s001.pdf]

Supplementary Information (SI) for Nanoscale Advances.
This journal is © The Royal Society of Chemistry 2026
Supplementary Information (SI) for Nanoscale Advances.
This journal is © The Royal Society of Chemistry

Supplementary Material

Caenorhabditis elegans in Nanosafety Research: Dual effect of *Escherichia coli* ecocorona formation on single and combined toxicity of graphene oxide

Francine Côa,[a,b,d,*] Mathias Strauss,[a] Cassio Hamilton Abreu-Junior,[b] Fabrício de Souza Delite,[a] Daiana Silva de Ávila,[c] Beatriz Santana Borges,[a] Rodrigo Villares Portugal,[a] Iseult Lynch,[d.e*] and Diego Stéfani Teodoro Martinez [a,b,*]

[a] Brazilian Nanotechnology National Laboratory (LNNano), Brazilian Center for Research in Energy and Materials (CNPEM), Campinas, São Paulo, Brazil

[b] Center for Nuclear Energy in Agriculture (CENA), Universidade de São Paulo (USP), São Paulo, Brazil

[c] Grupo de Pesquisa em Bioquímica e Toxicologia em Caenorhabditis elegans (GBToxCe), Universidade Federal do Pampa (UNIPAMPA), Uruguaiana, Rio Grande do Sul, Brazil

[d] School of Geography, Earth and Environmental Sciences, University of Birmingham, Edgbaston, B15 2TT Birmingham, United Kingdom

[e] Centre for Environmental Research and Justice, University of Birmingham, Edgbaston, B15 2TT Birmingham, United Kingdom

*Corresponding authors: francinecoa@gmail.com; i.lynch@bham.ac.uk; diego.martinez@lnnano.cnpem.br,

1. Experimental section

Escherichia coli culture and lysis

OP50 *Escherichia coli* was transferred from Luria-Broth (LB) solid medium with a sterile inoculating loop to sterile LB liquid medium (6 sterile Erlenmeyer flasks of 500 mL) and incubated in an orbital shaker (TE-4200, Tecnal) at 100 rpm and 37 °C. After 14 h, bacterial suspensions were transferred to 50 mL centrifuge tubes and pelleted (Centrifuge 5810R, Eppendorf) at 5,000 rpm, 4 °C for 5 min. Pellets were rinsed with phosphate buffer saline (PBS) and centrifuged again. Supernatants were discarded, and pellets were kept frozen until the extraction. Pellets (~3 g) were resuspended in 100 mL sterile PBS solution, and this suspension was sonicated in an ultrasonic tip processor (Model Gex 400, Acil Weber) operated at 40 W and controlled temperature (10 °C) for 10 min to promote the extraction of the intra-cellular compounds of bacteria. The suspension was centrifuged in 50 mL centrifuge tubes at 10,000 rpm, 4 °C for 10 min. Supernatants were collected and filtered through a 0.22 µm syringe filter to obtain only the soluble compounds from *E. coli* lysed. Proteins were quantified by Bradford assay (Lot #SLBV5669, Sigma-Aldrich) (**Figure S1**) at 595 nm using a microplate reader UV-Vis spectrophotometer (Multiskan TM GO, ThermoScientific) and the protein concentration in suspension was adjusted at 1.0 mg L^{-1} for further use.

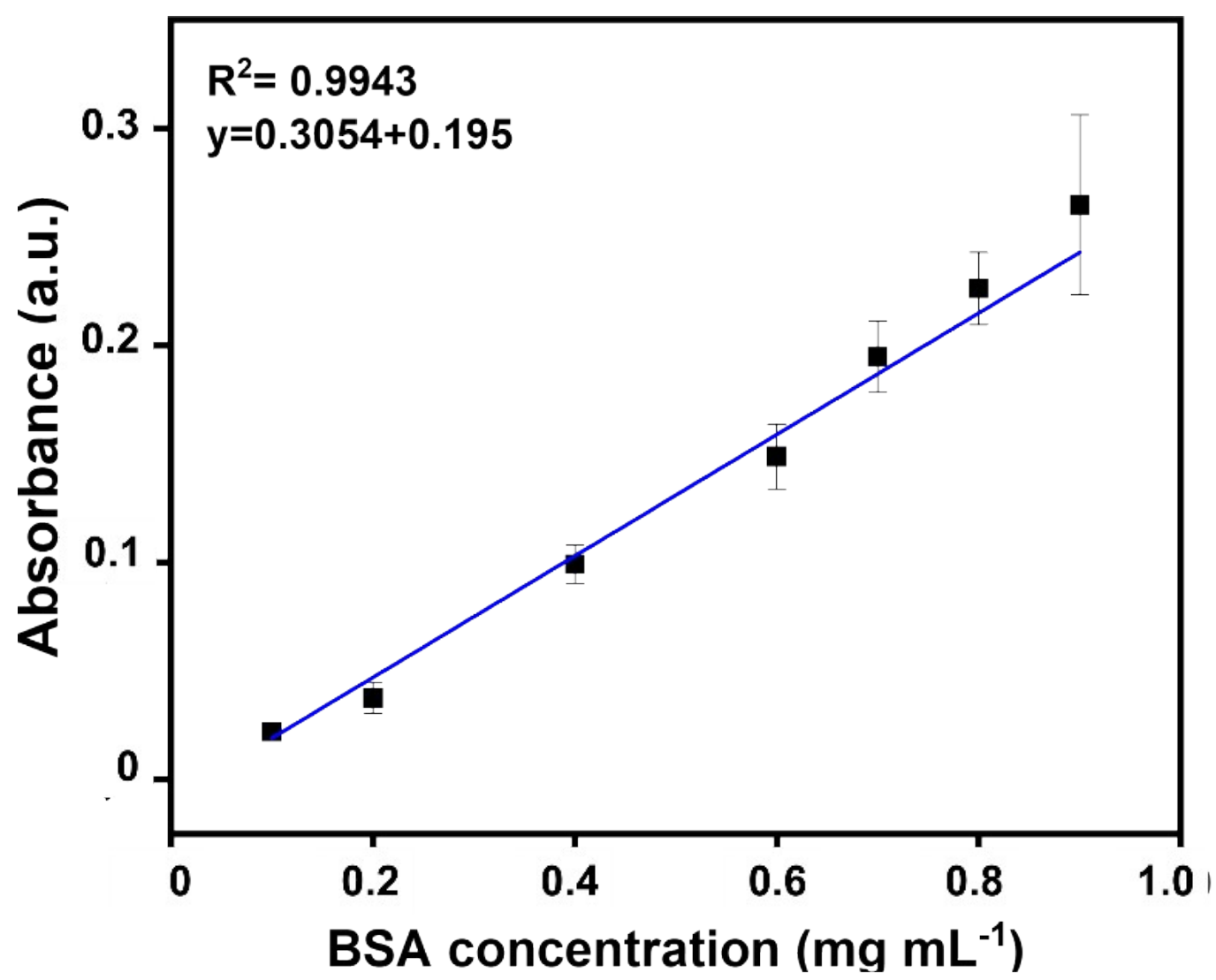

Fig. S1. Calibration curve of Bradford assay in PBS from absorbance (at 595 nm). The straight line is a linear fit of the data.

Characterization of graphene oxide with and without corona

The surface roughness and thickness features of GO and EC@GO were evaluated by atomic force microscopy (AFM). Images were obtained in a tapping mode on a MultiMode VIII microscope with NanoScope V controller module (Bruker). Silicon tips (NCHR, BudgetSensors) with a nominal resonance frequency of 300 kHz and a nominal force constant of 40 N m^{-1} were used. Images were acquired at a scan rate of 1 Hz over scan areas of 5 x 5 µm.

For AFM sample preparation, 5 µL of GO and EC@GO (10 mg L^{-1}) were dropped onto freshly cleaved mica substrates (2 x 2 cm) and dried in an acrylic chamber under nitrogen flow prior to AFM imaging. Average height profiles were acquired from 10 different flakes, and surface roughness was calculated as the root-mean-square (RMS) roughness over equivalent flake areas (12,808 nm^2). Images were processed on Gwyddion software (2.5 version). Plane leveling and background correction were applied prior to quantitative analysis.

X-ray photoelectron spectroscopy (XPS) was applied to evaluate the surface elemental composition of GO and EC@GO. A K-Alpha System (Thermofischer Scientific, USA) was operated with Al Kα X-rays and charge compensation. Survey spectra were collected with 400 μm spatial resolution at five different areas per sample using a pass energy of 50 eV. Data were processed with Thermo Avantage software (version 5.957).

Functional groups present on GO and EC@GO surfaces were evaluated by ATR-FTIR using an IRSpirit-L (Shimadzu). This was performed in the frequency range from 500 to 4000 cm^{-1}, with 2 cm^{-1} resolution and a total of 150 scans.

Sodium dodecyl sulfate-polyacrylamide gel electrophoresis (SDS-PAGE) was performed to separate the proteins adsorbed by GO by molecular weight. Bound proteins were removed from GO by adding 3xSDS-loading buffer (Lot#B77035, New England Biolabs) and 1:10 volume of 1.25M DTT for 3 min at 99 °C. Then, 15 μL of the removed proteins were loaded onto an SDS-PAGE (4% stacking gel and 15% resolving gel). Positive control was also loaded with all of the soluble proteins from *E. coli* lysis. Three independent samples were analysed, and the gel run included a color-prestained protein standard (Lot 0071601, New England BioLabs). After electrophoresis, separated proteins were stained with silver nitrate.

For LC-MS/MS analysis, gels from SDS-PAGE analyses were stained with Coomassie brilliant blue. Protein bands were cut and subjected to protein digestion and peptide extraction according to the procedure of Shevchenko et al. (1996).[1] Peptides were then purified in StageTips C18, dried in a vacuum concentrator and reconstituted in 20 μL of formic acid (0.1%), following the method described by Carnielli et al. (2018).[2]

Aliquots of 4.5 μL were analysed on an LTQ Orbitrap Velos mass spectrometer (Thermo Fisher Scientific, Waltham, MA, USA) connected to the EASY-nLC II system (Proxeon Biosystem, West Palm Beach, FL, USA) via a Proxeon nanoelectrospray ion

source. Peptides were separated using a PicoFrit analytical column (20 cm x ID 75 µm, 5 µm particle size, New Objective) with a gradient of 2–90% acetonitrile in 0.1% formic acid at a flow rate of 300 nL/min. The nanoelectrospray voltage was maintained at 2.2 kV, and the source temperature was set to 275 °C. The instrument operated in data-dependent acquisition mode. The full scan MS spectra (m/z 300–1600) were collected in the Orbitrap analyser after accumulation to a target value of 1×10^6. The Orbitrap resolution was set to 60,000, and the 20 most intense peptide ions with charge states of 2 or higher were sequentially isolated to a target value of 5,000 and fragmented in the linear ion trap using low-energy collision induced dissociation (CID) with a normalized collision energy of 35%. MS/MS events were triggered at a signal threshold of 1,000 counts. Dynamic exclusion was activated with a size list of 500, an exclusion duration of 60 seconds, and a repeat count of 1. An activation q-value of 0.25 and an activation time of 10 ms were applied.

Proteins were identified using MaxQuant v.1.5.8 against the UniProt *E-coli* Protein Database. Carbamidomethylation was used as a fixed modification, while N-terminal acetylation and methionine oxidation were set as variable modifications. The analysis allowed one trypsin-missed cleavage, with a precursor mass tolerance of 10 ppm and a fragment ion tolerance of 1 Da for protein identification. A maximum 1% false discovery rate was applied at both peptide and protein levels, using a reverse target-decoy database strategy, where reverse peptide sequences served as decoy entries. Bioinformatics analyses were conducted using the Perseus software (version 1.6), with data undergoing logarithmic transformation and filtering to exclude proteins with reversed sequences or those identified by only a modified peptide. Statistical analyses, including Student's t-test, were performed using Perseus, and Venn diagrams were generated using the Interactive Venn website (http://www.interactivenn.net/).

2. Results

2.1 Characterization of *Escherichia coli* ecocorona formation

As part of the characterization of *E. coli* ecocorona formation, atomic force microscopy (AFM) was employed to measure the thickness of GO and EC@GO flakes. The resulting height data were visualized using boxplots, enabling visualization of the distribution and variability of thickness values for each sample.

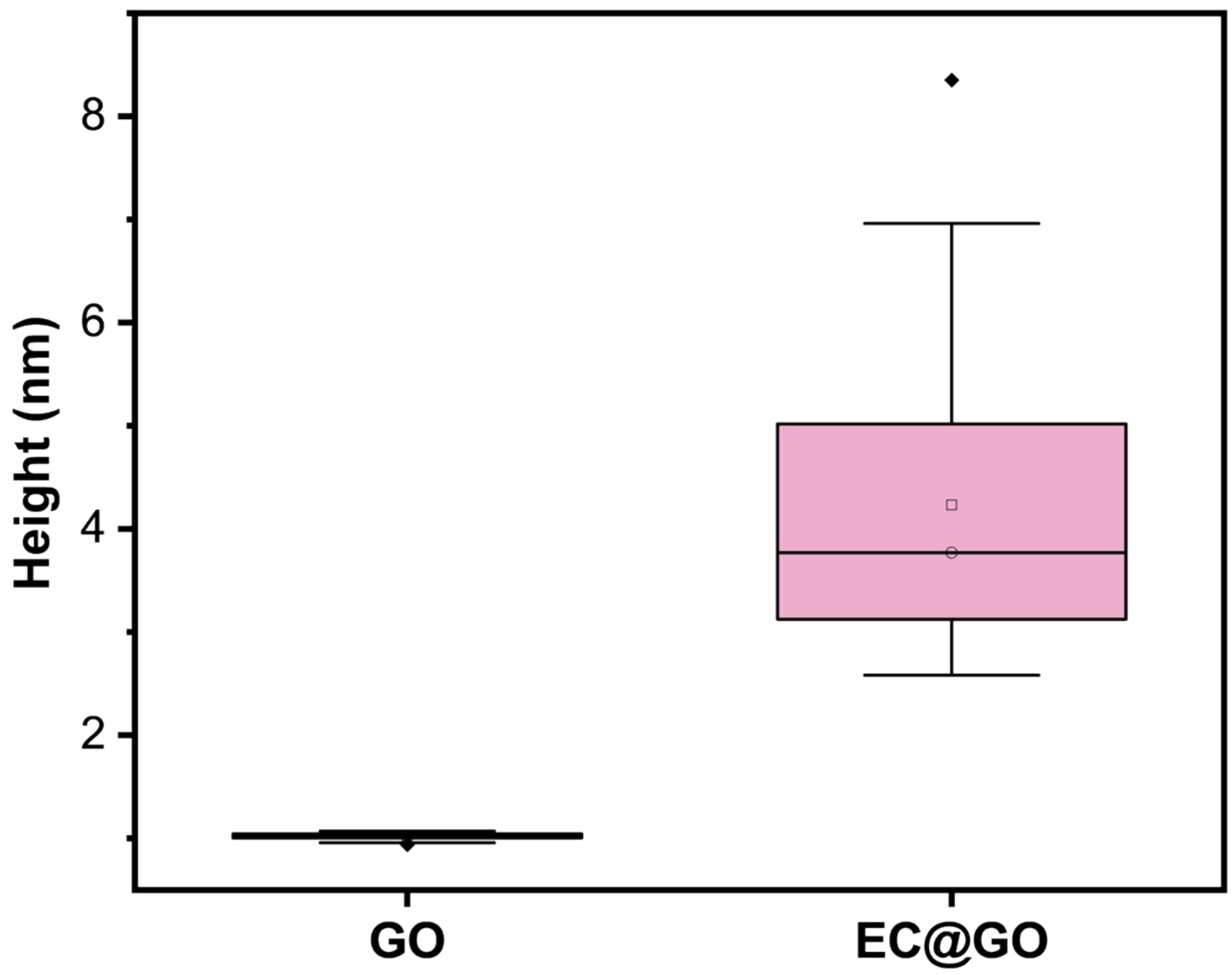

Fig. S2. Boxplot of AFM height distribution for GO and EC@GO samples. Thickness values were obtained from 20 independent flakes per condition.

To further characterize the physicochemical changes associated with ecocorona formation, the surface charge of GO was evaluated by zeta potential measurements. The zeta potential values of GO before and after ecocorona formation were measured in NaCl (0.1 mM) and EPA medium, enabling comparison of surface charge changes associated with ecocorona formation and medium composition, as presented in **Table S1**.

Table S1. Zeta potential values (mean±standard deviation) of GO before and after ecocorona formation in NaCl (0.1 mM) and EPA media.

Sample	NaCl (mV)	EPA (mV)
**GO**	**-48.7±1.2**	**-21.2±0.9 mV**
**EC@GO**	**-31.5±0.8**	**-22.1±0.4 mV**

To elucidate the composition of the *E. coli*-derived ecocorona formed on the graphene oxide surface, proteomic analysis (LC-MS/MS) was performed. The full proteomic datasets for *E. coli* lysate and the EC@GO fraction, including the complete list of identified proteins and associated analytical information, is provided in annex in **Table S2.**

Table S2 (annex). Excel file containing proteomic data (LC-MS/MS) of *E. coli* lysate and the *E. coli* ecocorona associated to the graphene oxide surface, including the complete list of identified proteins and corresponding analytical data.

In addition, during data interpretation, proteins identified in the lysate but not adsorbed onto the graphene oxide surface were separated and are presented below in **Table S3**.

Table S3. List of the 48 proteins identified in the total proteins but not adsorbed by GO (excluded from the ecocorona) according to the LC-MS/MS proteomic characterization.

Protein ID	MW (kDa)	Description	Organism	pI	Functional category	Function
P0AG81	48.44	Sn-glycerol-3-phosphate-binding periplasmic protein UgpB	*E. coli* O157:H7	6.34	Transport and periplasmic binding proteins	Part of the ABC Transporter complex UgpBAEC involved in sn-glycerol-3-phosphate (G3P) import
P09551	27.99	Lysine/arginine/ornithine-binding periplasmic protein	*E. coli* (strain K12)	5.63	Transport and periplasmic binding proteins	Part of the ABC transporter complex HisPMQ-ArgT
P0AAF6	27.02	Arginine transport ATP-binding protein ArtP	*E. coli* (strain K12)	6.32	Transport and periplasmic binding proteins	Part of the ABC transporter complex ArtPIQMJ involved in arginine transport
P0AD96	39.07	Leu/Ile/Val-binding protein	*E. coli* (strain K12)	5.57	Transport and periplasmic binding proteins	Component of the leucine, isoleucine, valine, (threonine) transport system
P0AEU0	28.48	Histidine-binding periplasmic protein	*E. coli* (strain K12)	5.47	Transport and periplasmic binding proteins	Part of the ABC transporter complex HisPMQJ
P0AFM2	36.02	Glycine betaine/proline betaine-binding periplasmic protein	*E. coli* (strain K12)	5.97	Transport and periplasmic binding proteins	Part of the ProU ABC transporter complex
P23847	60.29	Dipeptide-binding protein	*E. coli* (strain K12)	6.27	Transport and periplasmic binding proteins	Part of the ABC transporter DppABCDF involved in dipeptide transport
P30859	26.93	Putative ABC transporter arginine-binding protein 2	*E. coli* (strain K12)	5.82	Transport and periplasmic binding proteins	Part of the ABC transporter complex ArtPIQMJ involved in arginine transport
P30860	26.82	ABC transporter arginine-binding protein 1	*E. coli* (strain K12)	6.83	Transport and periplasmic binding proteins	Part of the ABC transporter complex ArtPIQMJ involved in arginine transport
P39265	32.91	D-allose-binding periplasmic protein	*E. coli* (strain K12)	6.43	Transport and periplasmic binding proteins	Part of the binding-protein-dependent transport system AlsBAC for D-allose
Q46863	60.69	Probable deoxycholate-binding periplasmic protein YgiS	*E. coli* (strain K12)	6.47	Transport and periplasmic binding proteins	Probably part of a deoxycholate transport system
O07380	55.46	Di-/tripeptide transporter	*Lactobacillus helveticus*	9.19	Transport and periplasmic binding proteins	Proton-dependent uptake of di or tri-peptides

Protein ID	MW (kDa)	Description	Organism	pI	Functional category	Function
Q1RFJ9	41.15	Iron uptake system component EfeO	*E. coli (strain UTI89/UPEC)*	5.03	Iron and metal metabolism	Involved in Fe^{2+} uptake
Q0TEV7	11.55	Iron-binding protein IscA	*E. coli* O6:K15:H31	4.76	Iron and metal metabolism	Transfer iron-sulfur clusters to apo-ferredoxin
B7LKB2	20.13	Co-chaperone protein HscB	*E. fergusonii, E. coli* O8	5.06	Iron and metal metabolism	Involved in the maturation of iron-sulfur cluster-containing proteins
P0A9R4	12.33	2Fe-2S ferrodoxin	*E. coli* (strain K12)	4.43	Iron and metal metabolism	Ferredoxin are iron-sulfur proteins that transfer electrons in a wide variety of metabolic reactions
P0ABD3	18.49	Bacterioferritin	*E. coli* (strain K12)	4.70	Iron and metal metabolism	Iron storage protein
Q8XBY2	12.21	Cation efflux system protein CusF	*E. coli* O157:H7	8.01	Iron and metal metabolism	Part of a cation efflux system that mediates resistance to copper and silver
Q8CVW3	18.16	Ecotin	*E. coli* O6:H1	6.59	Stress response and protein folding	General inhibitor of pancreatic serine proteases
Q1R8B1	21.85	Protein GrpE	*E. coli* (strain UTI89)	4.65	Stress response and protein folding	Participates actively in the response to hyperosmotic
P0AFH8	21.07	Osmotically-inducible protein Y	*E. coli* (strain K12)	6.37	Stress response and protein folding	Response to osmotic stress
P0A4L5	23.11	Thiol:disulfide interchange protein DsbA	*E. coli* O6:H1	6.00	Stress response and protein folding	Required for disulfide bond formation in some periplasmic proteins
B7LLQ1	19.9	Adenine phosphoribosyltransferase	*Shigella flexneri*, *E. coli* O157:H7	5.12	Metabolism and biosynthesis	Glycosyltransferase
Q8FE66	13.82	Glycine cleavage system H protein	*E. coli* O6:H1	3.98	Metabolism and biosynthesis	Catalyzed the degradation of glycine
Q83IN1	39.64	N-acetylneuraminate epimerase	*Shigella flexneri*	6.78	Metabolism and biosynthesis	Converts alpha-N-acetylneuranimic acid (Neu5Ac) to the beta-anomer
P32669	23.55	Fructose-6-phosphate aldolase 2	*E. coli* (strain K12)	5.05	Metabolism and biosynthesis	Catalyzes the reversible formation of fructose 6-phosphate
P77258	39.51	N-ethylmaleimide reductase	*E. coli* (strain K12)	5.87	Metabolism and biosynthesis	Involved in the degradation of toxic compounds

Protein ID	MW (kDa)	Description	Organism	pI	Functional category	Function
P78218	17.50	Dihydrofolate reductase type 15	*E. coli*	5.60	Metabolism and biosyntheis	Key enzyme in folate metabolism
P56262	29.42	Putative carboxymethylenebutenolidase	*E. coli* (strain K12)	5.63	Metabolism and biosynthesis	-
P0A82	10.23	DNA-directed RNA polymerase subunit omega	*E. coli* O157:H7	4.87	Gene expression and regulation	Promotes RNA polymerase assembly
P31802	23.57	Nitrate/nitrite response regulator protein NarP	*E. coli* (strain K12)	4.82	Gene expression and regulation	Activates the expression of the nitrate reductase
P33012	18.08	DNA gyrase inhibitor	*E. coli* (strain K12)	4.61	Gene expression and regulation	Inhibits the supercoiling activity of DNA gyrase
B5YZ66	9.40	Cell division protein ZapB	*E. coli* O157:H7	4.69	Cell structure, envelope and motility	Non-essential, abundant cell division factor that is required for proper Z-ring formation
P0ACV6	26.55	Murein peptide amidase A	*E. coli* (strain K12)	5.62	Cell structure, envelope and motility	Involved in muropeptide degradation
P31433	62.27	AsmA family protein YicH	*E. coli* (strain K12)	5.72	Cell structure, envelope and motility	-
P33235	57.93	Flagellar hook-associated protein 1	*E. coli* (strain K12)	4.48	Cell structure, envelope and motility	Structural molecule activity
P75937	42.04	Flagellar hook protein FlgE	*E. coli* (strain K12)	4.46	Cell structure, envelope and motility	Cellular component (cytosol)
P27668	55.89	Signal transduction histidine-protein kinase/phosphatase UhpB	*Salmonella typhimurium*	7.42	Signal transduction	Part of the UhpABC signaling cascade
P76330	64.28	Probable diguanylate cyclase DgcQ	*E. coli* (strain K12)	6.78	Signal transduction	Catalyzes the synthesis of cyclic-di-GMP (c-di-GMP) via the condensation of 2 GTP molecules
P05458	107.70	Protease 3	*E. coli* (strain K12)	5.82	Proteolysis and peptide metabolism	Endopeptidase that degrades small peptides of less than 7 kDa
Q1R3S5	24.57	Peptidase E	*E. coli* (strain UTI89)	5.73	Proteolysis and peptide metabolism	Hydrolyzes dipeptides containing N-terminal aspartate residues
P76256	25.18	tRNA threonylcarbamoyladenosine biosynthesis protein TsaB	*E. coli* (strain K12)	5.01	tRNA modification	Formation of a threonylcarbamoyl group on adenosine
O07380	55.46	Di-/tripeptide transporter	*Lactobacillus helveticus*	9.19	Transport	Proton-dependent uptake of di or tri-peptides

Protein ID	MW (kDa)	Description	Organism	pI	Functional category	Function
B7UHV7	21.23	UPF0149 protein YgfB	*E. coli* O127:H6	4.19	Uncharacterized protein	-
P24178	13.60	Protein YffB	*E. coli* (strain K12)	6.14	Uncharacterized protein	-
P51024	19.92	Uncharacterized protein YaiL	*E. coli* (strain K12)	7.72	Uncharacterized protein	Cellular component (cytosol)
P0AF56	25.07	Sel1-repeat-containing protein YjcO	*E. coli* (strain K12)	4.77	Uncharacterized protein	-
O4825	6.60	Protein NinF	*Enterobacteria phage* H19B	4.24	Uncharacterized protein	-

pI: predicted isoelectric point, MW: molecular weigh

In the next step, proteins exclusively identified in the EC@GO fraction, and not detected in the lysate dataset, were separated during data interpretation and are presented below in **Table S4,** likely reflecting enrichment of low-abundance proteins upon adsorption onto the graphene oxide surface.

Table S4. List of the 24 proteins identified exclusive in the ecocorona of graphene oxide (but with too low abundance to be detected in the total lysate) by LC-MS/MS proteomic characterization.

Protein ID	MW (kDa)	Description	Organism	pI	Function
Q9JMR7	10.748	Uncharacterized protein YuaX	*E. coli* K12	4.49	-
P0ADW5	14.961	Z-ring associated protein G	*E. coli* O157:H7, O6:H1, K12	5.63	Cell division
P33355	17.973	Uncharacterized protein YehS	*E. coli* K12	9.20	-
P0A832	18.269	SsrA-binding protein	*E. coli* K12	9.90	RNA-binding, trans-translation
P0AC52	19.254	Zinc uptake regulation protein	*E. coli* K12, *Shigella flexneri*	5.97	DNA-binding, transcription
P0A809	22.066	Holliday junction branch migration complex subunit RuvA	*E. coli* K12	5.72	Holliday junction helicase complex, ATP-binding
P0ADT9	23.076	Uncharacterized protein YgiM	*E. coli* O6:H1, O157:H7, *Shigella flexneri*	9.10	Cellular component (membrane)
P0C0R7	23.335	Ribosomal RNA large subunit methyltransferase E	*E. coli* K12	9.44	Catalytic activity
P0DMC9	23.516	Transcriptional regulatory protein RcsA	*E. coli* K12	9.28	Transcription regulator complex
P32719	26.109	D-allulose-6-phosphate 3-epimerase	*E. coli* K12	5.34	Isomerase, carbohydrate metabolism
P37769	27.070	2-dehydro-3-deoxy-D-Gluconate 5-dehydrogenase	*E. coli* K12	5.24	Oxireductase activity
Q8FBT8	27.417	Phosphate-specific transport system accessory protein PhoU	*E. coli* O6:H1, *Shigella flexneri*	5.01	Phosphate regulon
P39298	27.588	Esterase YjfP	*E. coli* K12	5.19	Hydrolase activity, proteolysis, protein homodimerization activity
Q8FBI6	28.892	Sec-independent protein translocase protein TatC	*E. coli* O6:H1	5.25	Protein transport
P00638	34.502	Exonuclease	Escherichia phage T7	4.93	DNA-binding, exonuclease activity
P0ACP6	36.422	HTH-type transcriptional regulator GntR	*E. coli* K12	6.41	DNA-binding, transcription
P17443	37.812	UDP-N-acetylglucosamine--N-acetylmuramyl-(pentapeptide) pyrophosphoryl-undecaprenol N-acetylglucosamine transferase	*E. coli* K12	9.70	Cell wall formation
P25539	40.338	Riboflavin biosynthesis protein RibD	*E. coli* K12	7.22	5-amino-6-(5-phosphoribosylamino) uracil

Protein ID	MW (kDa)	Description	Organism	pI	Function
					reductase activity
P75863	40.644	Uncharacterized protein YcbX	*E. coli* K12	7.58	Catalytic activity, pyridoxal phosphate binding
P27303	42.736	Multidrug export protein EmrA	*E. coli* K12	9.33	Transport
P26648	51.858	Cell division protein FtsP	*E. coli* K12	5.94	Cell division
P39006	56.594	Phosphatidylserine decarboxylase proenzyme 1, mitochondrial	*Saccharomyces cerevisiae*	9.18	Decarboxylase, lyase, lipid biosynthesis, and lipid metabolism
A1A9B7	70.695	Macrolide export ATP-binding/permease protein MacB 1	*E. coli* O1:K1	5.95	Translocase, ATP-binding, nucleotide-binding
P62517	96.936	Glucans biosynthesis glucosyltransferase H	*E. coli* K12	8.87	Glycosyl transferase

pI: predicted isoelectric point, MW: molecular weight.

To further compare protein abundance profiles, the most abundant proteins identified across the datasets were selected and are presented in **Table S5**. These proteins were grouped into clusters corresponding to total proteins (i.e., *E. coli* lysate) and the EC@GO fraction. Their abundance profiles are visualized in the heat map shown in **Fig. S3**.

Table S5. Excel file containing list of the 567 most abundant proteins in control (*E. coli* lysed - total proteins) and eluted from GO ecocorona.

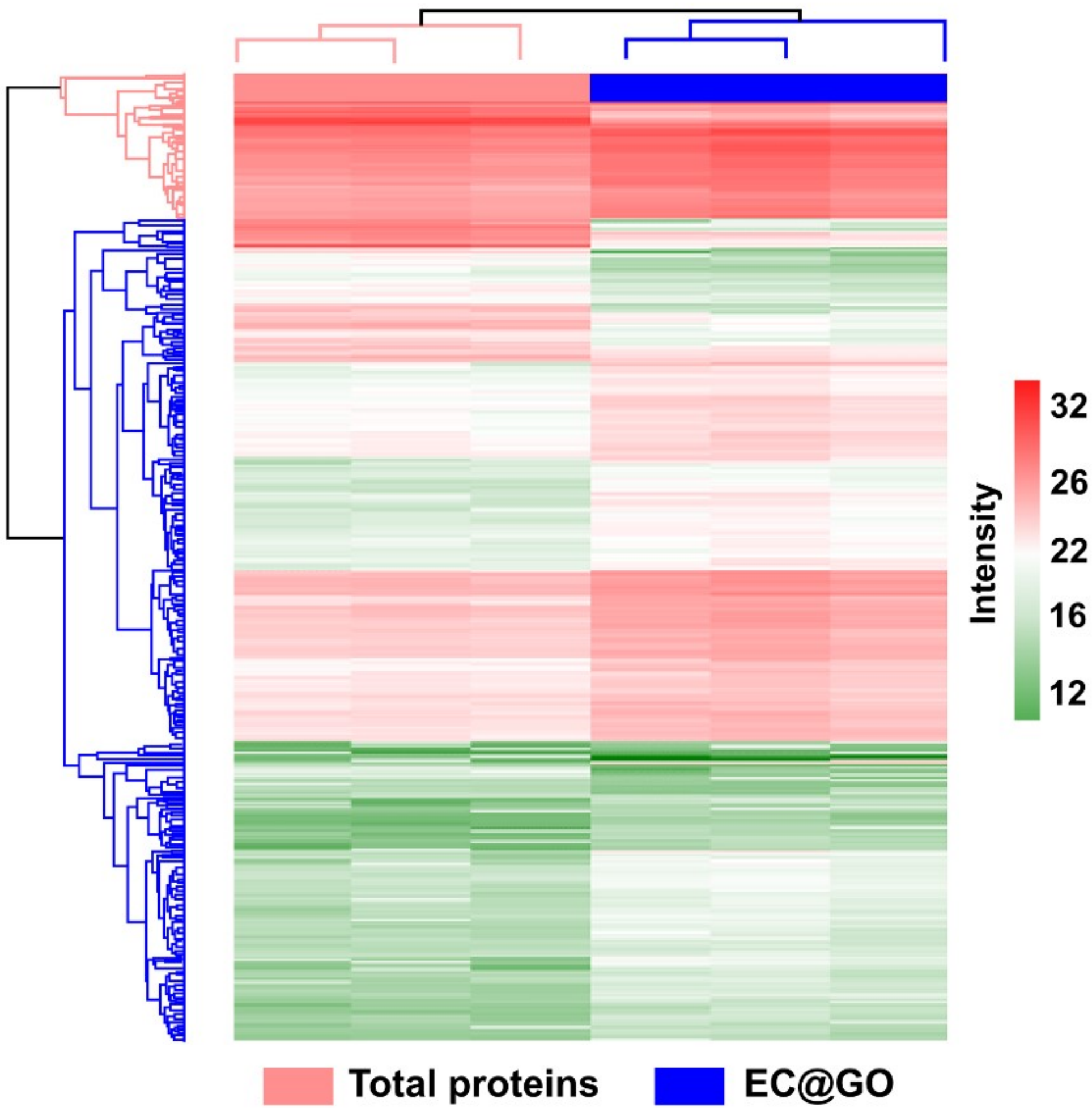

Fig. S3. Heat map of the top 567 most abundant proteins identified in *E. coli* lysate and EC@GO samples. Proteins are hierarchically clustered based on their relative abundance profiles. Each row represents a single protein, and each column corresponds to an

individual replicate (n = 3 per condition). Color intensity reflects normalized protein abundance (low to high, green to red). Samples are grouped into two conditions: *E. coli* lysate (pink) and EC@GO (blue).

2.2. The determining role of the *E. coli* ecocorona in mitigating GO toxicity

In this study, confocal Raman spectroscopy was employed to investigate the internalization and biodistribution of GO and EC@GO in *C elegans*. Complementary bright-field images of nematodes exposed to GO, EC@GO, GO+Ag^+ and EC@GO+Ag^+ containing treatments are provided in **Fig. S4**. The Raman spectra of the studied materials are presented in **Fig. S5**, followed by representative spectra acquired from control (unexposed) and exposed organisms (**Fig. S6**).

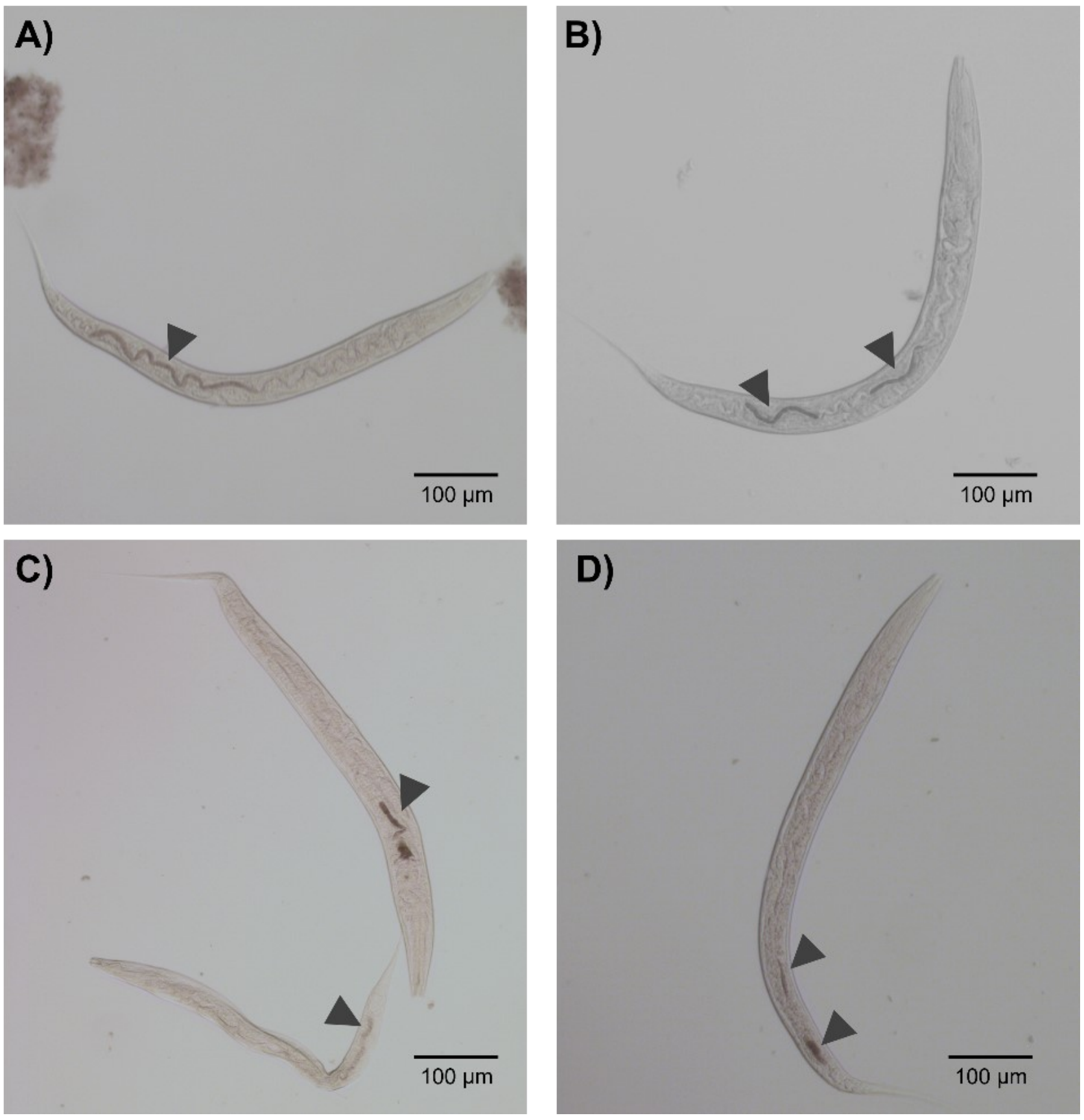

Fig. S4. Representative bright-field images of *C. elegans* exposed to GO (A), EC@GO (B), GO+Ag^+ (C), and EC@GO+Ag^+ (D). Arrowheads indicate dark aggregates observed

within the intestinal lumen. These images are provided as complementary visual information to the Raman analyses.

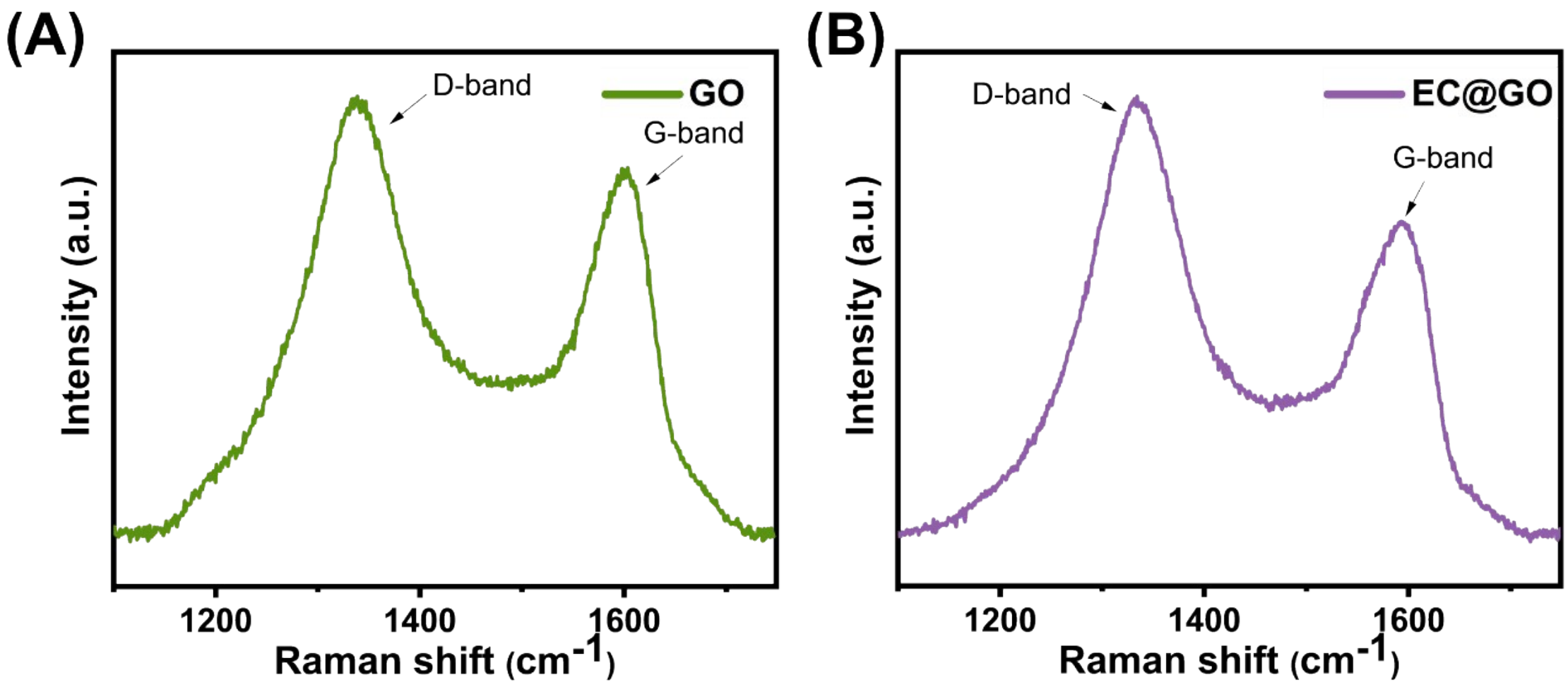

Fig. S5. Raman spectra of GO (A) and EC@GO (B), highlighting the characteristic D and G bands.

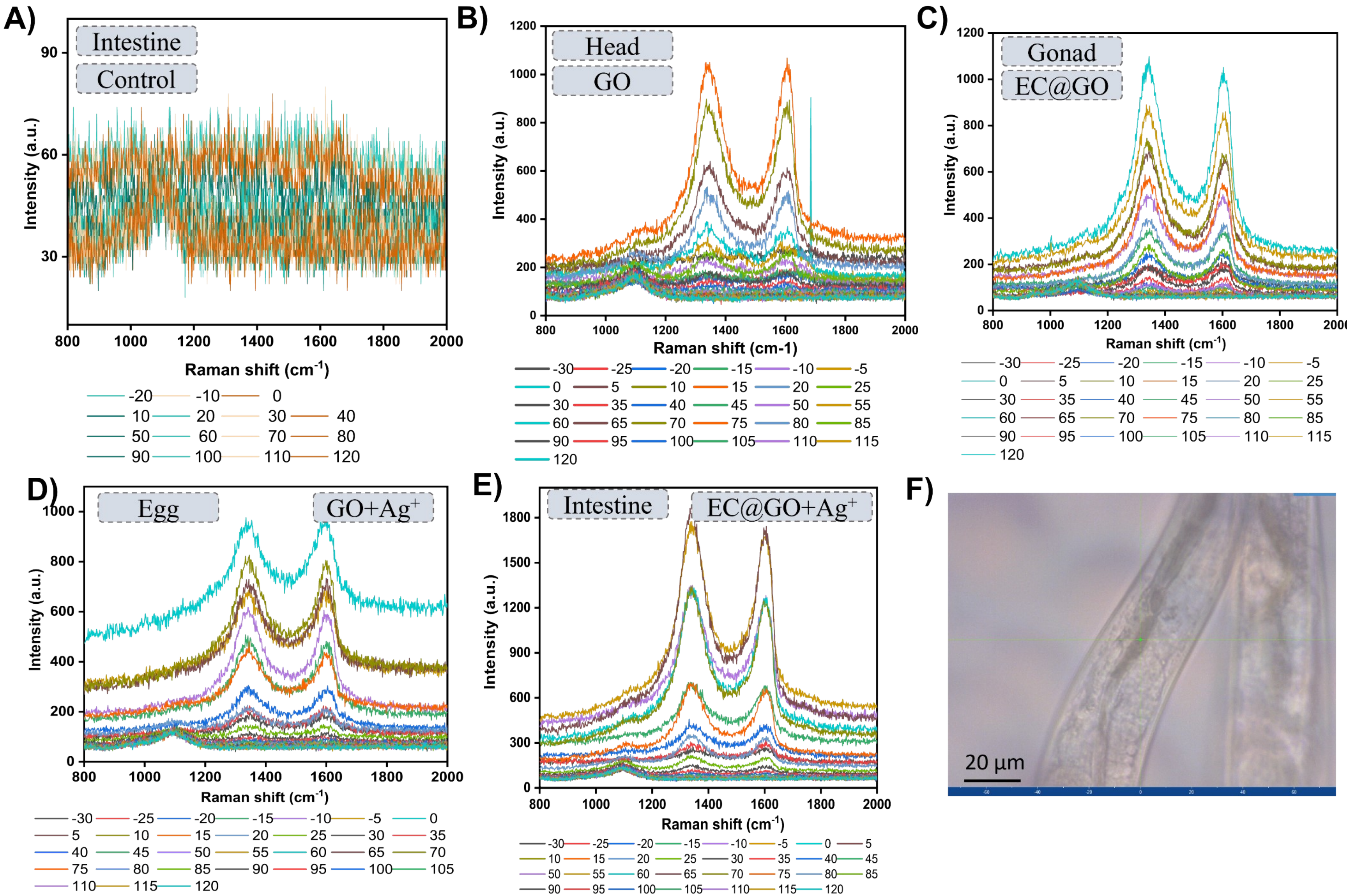
A)
Intestine
Control
Intensity (a.u.)
Raman shift (cm-1)
-20 -10 0 10 20 30 40 50 60 70 80 90 100 110 120
B)
Head
GO
Intensity (a.u.)
Raman shift (cm-1)
-30 -25 -20 -15 -10 -5 0 5 10 15 20 25 30 35 40 45 50 55 60 65 70 75 80 85 90 95 100 105 110 115 120
C)
Gonad
EC@GO
Intensity (a.u.)
Raman shift (cm-1)
D)
Egg
GO+Ag+
Intensity (a.u.)
Raman shift (cm-1)
E)
Intestine
EC@GO+Ag+
Intensity (a.u.)
Raman shift (cm-1)
F)
20 μm

Fig. S6. Representative Raman spectra acquired from different regions of *C. elegans* under distinct exposure conditions. A) Intestine region of control (unexposed) nematode. B) Head region of nematode exposed to GO. C) Gonad region of nematode exposed to EC@GO. D) Egg region of nematode exposed to GO+Ag^{+}. E) Intestine region of nematode exposed to EC@GO+Ag^{+}. F) Representative optical microscopy image acquired using the confocal Raman microscope, indicating one of the intestinal regions where the spectra were obtained.

Based on the evidence that both GO and EC@GO were internalized inside the nematode body, we investigated whether exposure to these materials affected the neurons. **Fig. S7** shows the effects of GO and EC@GO on *C. elegans* neurons, evaluated using the AML175 strain.

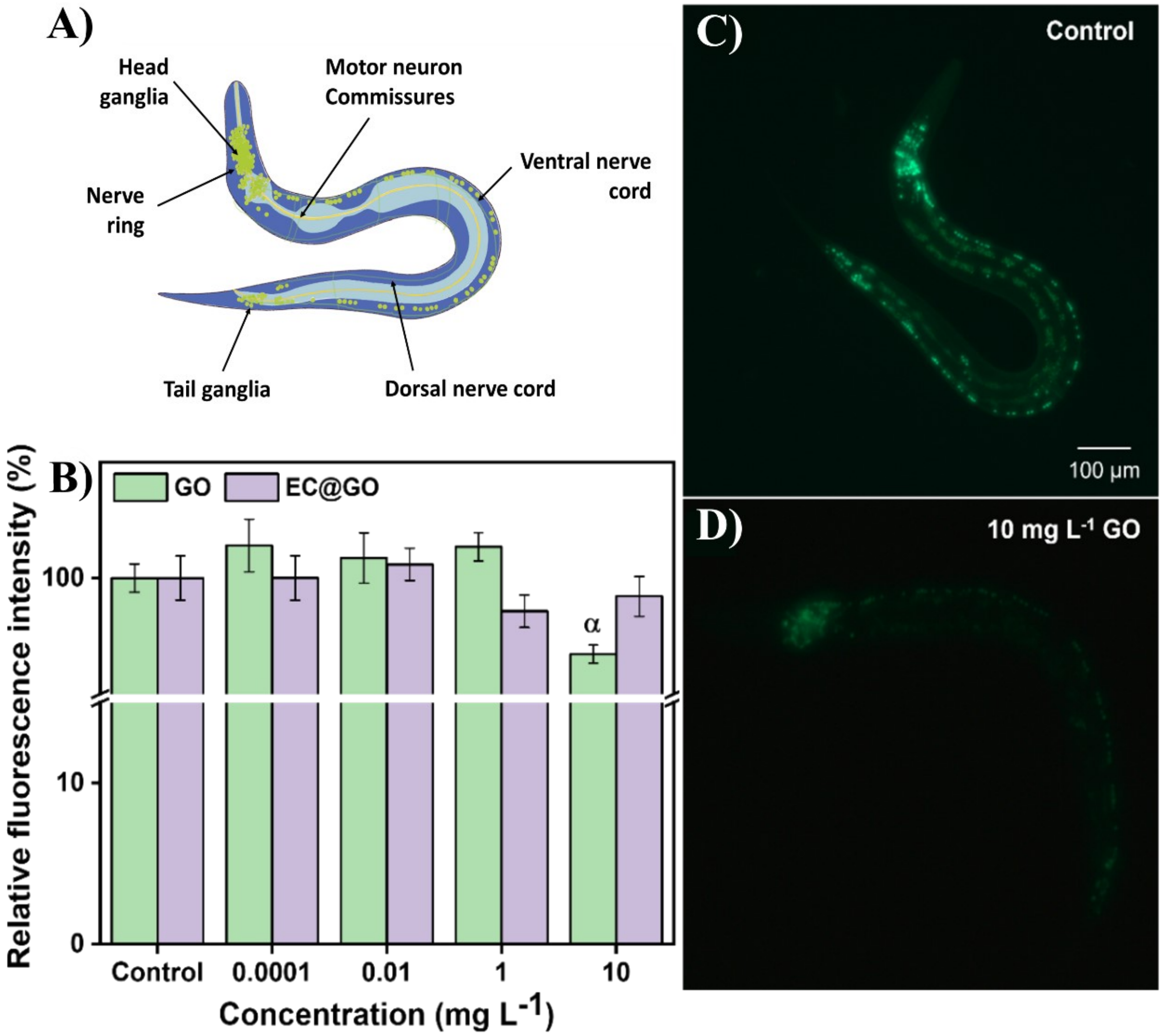

Figure S7. Effects of GO and EC@GO on *C. elegans* neurons evaluated in the AML175 strain. **A)** Diagram of *C. elegans* neuronal system. **B)** Effects of GO and EC@GO (0.0001 to 10 mg L^{-1}) on the *C. elegans* neuronal system. α indicates the treatments that are statistically different from the control group ($p<0.05$). **C)** Representative fluorescence images of unexposed nematode (control) and **D)** nematode exposed to 10 mg L^{-1} of GO.

Colloidal stability of GO in the presence of heavy metals during toxicity assays is a critical factor to be considered. Therefore, the behaviour of GO+Ag^{+} and EC@GO+Ag^{+} was evaluated in ultrapure water (UW) and EPA medium over 24 h. The results are presented in **Fig. S8.**

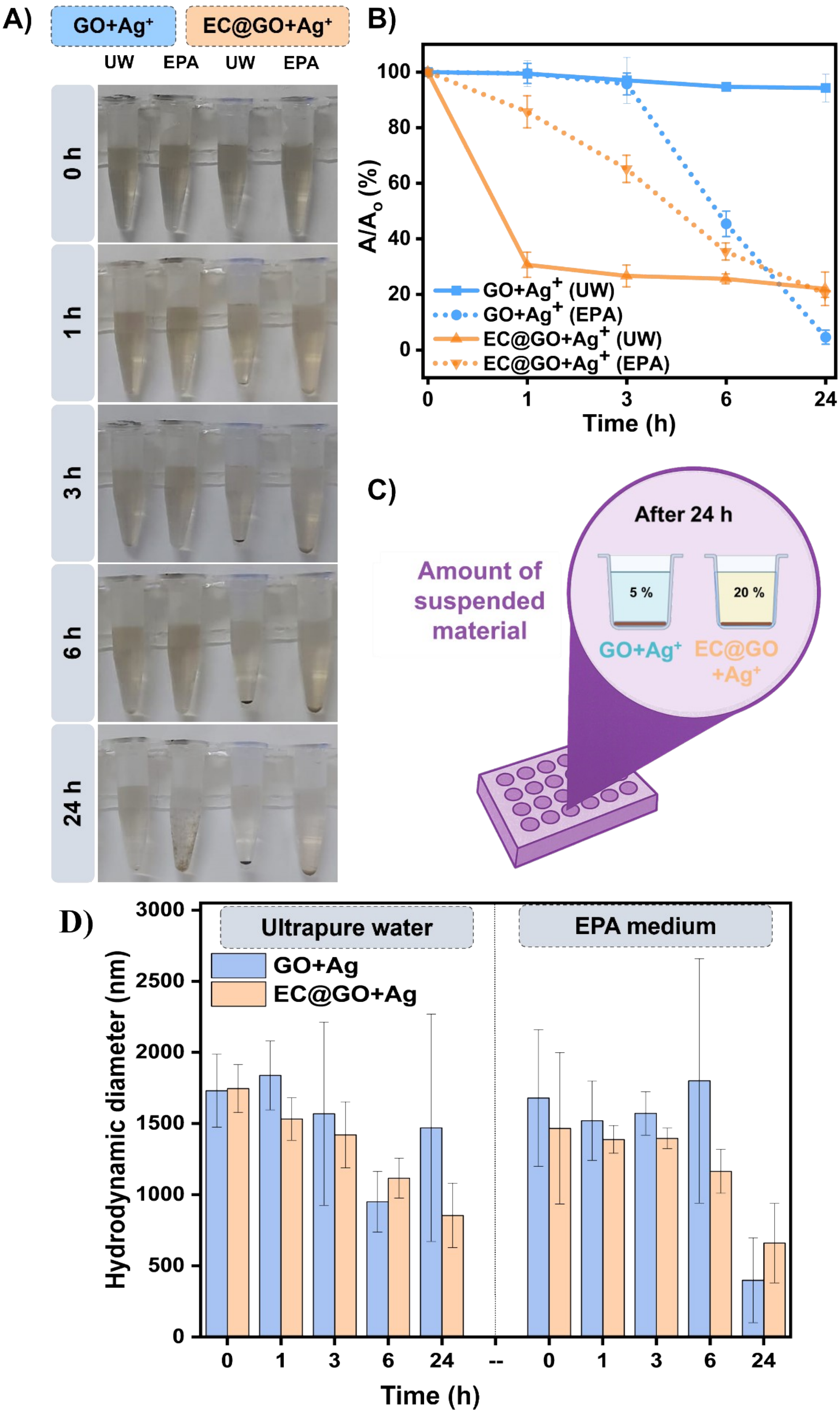
A)
GO+Ag+
EC@GO+Ag+
UW
EPA
UW
EPA
0 h
1 h
3 h
6 h
24 h
B)
A/Ao (%)
100
80
60
40
20
0
GO+Ag+ (UW)
GO+Ag+ (EPA)
EC@GO+Ag+ (UW)
EC@GO+Ag+ (EPA)
0
1
3
6
24
Time (h)
C)
After 24 h
Amount of suspended material
5 %
20 %
GO+Ag+
EC@GO +Ag+
D)
Ultrapure water
EPA medium
GO+Ag
EC@GO+Ag
Hydrodynamic diameter (nm)
3000
2500
2000
1500
1000
500
0
0
1
3
6
24
--
0
1
3
6
24
Time (h)

Figure S8. Colloidal stability of $GO+Ag^+$ and $EC@GO+Ag^+$ in ultrapure water (UW) and EPA medium. **A)** Visual inspection of the materials' colloidal behaviour over 24 h. **B)** Amount of suspended materials over 24 h measured by UV-Vis spectroscopy. **C)** Illustration of the amount of suspended materials after 24 h of analysis. **D)** Colloidal stability of GO and EC@GO at 10 mg L^{-1} by DLS.

References

1 A. Shevchenko, M. Wilm, O. Vorm and M. Mann, *Anal Chem*, 1996, **68**, 850–858.

2 C. M. Carnielli, C. C. S. Macedo, T. De Rossi, D. C. Granato, C. Rivera, R. R. Domingues, B. A. Pauletti, S. Yokoo, H. Heberle, A. F. Busso-Lopes, N. K. Cervigne, I. Sawazaki-Calone, G. V. Meirelles, F. A. Marchi, G. P. Telles, R. Minghim, A. C. P. Ribeiro, T. B. Brandão, G. de Castro, W. A. González-Arriagada, A. Gomes, F. Penteado, A. R. Santos-Silva, M. A. Lopes, P. C. Rodrigues, E. Sundquist, T. Salo, S. D. da Silva, M. A. Alaoui-Jamali, E. Graner, J. W. Fox, R. Della Coletta and A. F. Paes Leme, *Nat Commun*, 2018, **9**, 3598.
